# Physicochemical Characteristics and Flavor-Related Compounds of Fresh and Frozen-Thawed Thigh Meats from Chickens

**DOI:** 10.3390/foods11193006

**Published:** 2022-09-27

**Authors:** Farouq Heidar Barido, Hye-Jin Kim, Dong-Jin Shin, Ji-Seon Kwon, Hee-Jin Kim, Dongwook Kim, Hyo-Jun Choo, Ki-Chang Nam, Cheorun Jo, Jun-Heon Lee, Sung-Ki Lee, Aera Jang

**Affiliations:** 1 Department of Applied Animal Science, College of Animal Life Sciences, Kangwon National University, Chuncheon 24341, Korea; 2 Department of Agricultural Biotechnology, Center for Food and Bioconvergence, and Research Institute of Agriculture and Life Science, Seoul National University, Seoul 08826, Korea; 3 Poultry Research Institute, National Institute of Animal Science, Pyeongchang 25342, Korea; 4 Department of Animal Science and Technology, Sunchon National University, Suncheon 57922, Korea; 5 Division of Animal and Dairy Science, Chungnam National University, Daejeon 34134, Korea

**Keywords:** frozen-thawed, Korean native chickens, organoleptic properties, physicochemical, thigh meat

## Abstract

The physicochemical characteristics and flavor-related compounds of thigh meat derived from diverse Korean native chickens (KNC), namely Hanhyup No. 3 (HH3), Woorimatdag No 1 (WRMD 1), and Woorimatdag No 2 (WRMD 2), under fresh and frozen-thawed conditions were studied and compared with those of commercial broilers (CB). Regardless of the breed, KNC showed a higher (*p* < 0.05) percentage of linoleic and arachidonic acid. The highest proportion of docosahexaenoic acid was observed in WRMD 2. Despite having a higher collagen content, thigh meat derived from KNC maintained a similar texture profile in comparison to that of CB. The concentrations of most free amino acids (FAA), except for taurine, tryptophan, and carnosine, were higher in frozen-thawed meat than in fresh meat. Regarding volatile organic compounds (VOC), following freezing, the concentration of favorable VOCs increased in CB, but decreased in WRMD 1, suggesting a loss of pleasant flavor in frozen-thawed meat. This study indicated that changes in VOCs, including hydrocarbons (d-limonene, heptadecane, hexadecane, naphthalene, pentadecane, 3-methyl-, tridecane), esters (arsenous acid, tris(trimethylsilyl) ester, decanoic acid, ethyl ester, hexadecanoic acid, ethyl ester), alcohol (1-hexanol, 2-ethyl-), ketones (5,9-undecadien-2-one, 6,10-dimethyl-), and aldehydes (pentadecanal-, tetradecanal, tridecanal), may be a promising marker for distinguishing between fresh and frozen-thawed chicken thigh meat. These findings are of critical importance as preliminary data for developing high-quality chicken meat products.

## 1. Introduction

Consumer preferences for meat products are highly determined by a set of factors, including nutritional content, mouthfeel sensation, and safety guarantee for continuous consumption [1]. Among the available options, chicken meat is highly favored because of its high protein level, low saturated fat and cholesterol content, and relatively affordable cost. This has resulted in a steady increase in the demand for chicken meat. In Korea, the total domestic consumption of chicken meat reached 1.06 million metric tons in 2021 [2], of which commercial broiler (CB) chicken, Korean native chicken (KNC), and spent hen chicken made up the major proportions [3]. In 2012, the Korean government, through the Golden Seed Project, established a program to develop and commercialize the sustainable production of KNC to conserve highly valuable domestic animal sources and maintain genetic heterozygosity to avoid widespread diseases [4]. Supported by steadily increasing domestic needs, which encompassed more than 10% of the total consumption by 2019 [3], the provision of a supply of KNC has huge potential as primary stream revenue.

To date, among the highly bred KNC breeds, Hanhyup No. 3 (HH3), which is generated via cross-mating between KNC and economically supreme breeds, accounted for 80% of the market share of KNC in Korea and is classified as a premium chicken breed with a 150% higher price than CB [5]. Hence, the Woorimatdag No. 1 (WRMD 1) breed was developed to increase the taste quality and affordability of KNC. Studies have shown that this breed has notably higher taste-active compounds than CB, resulting in more intense taste profiles [6,7]. In addition, the contents of bioactive compounds, namely anserine, betaine, carnosine, carnitine, and creatine, are substantially higher in WRMD 1 than in CB, making the meat taste unique and preferred by Korean consumers [6]. Despite its advantageous characteristics, the low growth performance of WRMD 1 makes it difficult for its production to meet market demand. Therefore, the Woorimatdag No. 2 (WRMD 2) breed was developed as a commercially available KNC with increased growth performance and meat quality compared to WRMD 1 [5,8]. This breed is a result of cross-mating between Brown KNC males and Rhode Island Red females. To date, in-depth studies characterizing the physicochemical traits and organoleptic compounds of WRMD 1 and 2 are scarce.

In the poultry industry, two classifications of deboned meat are widely recognized: fresh meat, which is obtained by slaughtering and deboning a chicken in the same slaughterhouse, and frozen-thawed meat, which undergoes freezing and thawing following slaughter and deboning in the same slaughterhouse [9]. Although freezing is an accepted method for preserving the quality of meat proteins, frozen products are still of lower quality than fresh products [10,11]. Moreover, the thawing process triggers the release of proteases, lipases, and lysozomes from damaged cells, which consequently affects biochemical homeostasis [12]. Furthermore, the formation of ice crystals within meat largely affects the sensitive ultrastructure of proteins, and thawing causes the ice crystals to melt, thereby transferring the intracellular water to the extracellular area of meat, leading to excessive moisture loss, decreased texture profile, and protein denaturation and oxidations [11]. Frozen-thawed CB chicken thigh was reported to exhibit lower meat quality and higher total aerobic bacterial counts than its fresh counterpart, resulting in a notably lower overall organoleptic acceptability [9]. However, short-term frozen storage was reported to intensify the flavor profiles of yellow-feathered female chicken meat upon processing [13].

Considering the limited information available on various KNC breeds, especially WRMD 1 and WRMD 2, and on the effects of fresh and frozen-thawed conditions on chicken thigh, this study was performed to compare the physicochemical characteristics and organoleptic compounds of fresh and frozen-thawed thigh meat derived from KNC breeds. The results of this study provide pivotal information to advance the development and application of KNC as a raw material for meat products.

## 2. Materials and Methods

### 2.1. Chicken Samples

Three types of KNC (Hanhyup No.3, HH3; Woorimatdag No.1, WRMD1; and Woorimatdag No.2, WRMD2) were purchased from domestic meat shops (each *n* = 20) in Korea. The meats were then stored in the laboratory at 4 °C. Half of them were used as fresh samples, whereas the other half were directly frozen in a −18 °C freezer as frozen-thawed samples. Frozen chicken was thawed in a refrigerator at 4 °C for 16 h before the experiment. Boneless and skinless thigh meats were obtained from each group of chicken and used for the experiments.

### 2.2. Proximate Composition

The proximate composition of chicken thigh meat was evaluated using the official methods of analysis stipulated by the Association of Official Agricultural Chemists (AOAC, 1997) [14]. Moisture content was assessed by oven drying at 105 °C for 16 h. Crude protein content was analyzed using the Kjeldahl method with a conversion factor of 6.25. Crude fat was assayed by solvent extraction. Crude ash content was analyzed by burning the samples in a furnace at 550 °C for 12 h.

### 2.3. Phsyicochemical Composition 

The pH of chicken thigh was measured as follows: 10 g of meat was homogenized with distilled water (90 mL) for 15 s using a homogenizer (Polytron PT-2500E; Kinematica, Lucerne, Switzerland) according to the method of Kim et al. [15]. The pH value of the homogenate was determined using an Orion 230A pH meter (Thermo Fisher Scientific, Waltham, MA, USA).

The color of the chicken thigh meat was measured using a Chroma Meter CR-400 instrument (Minolta Co., Osaka, Japan), with the parameters CIE L* (lightness), CIE a* (redness), and CIE b* (yellowness). The chroma meter was calibrated using white plate references (Y = 93.60, x = 0.3134, and y = 0.3194).

The water-holding capacity (WHC) of the chicken thigh meat was evaluated as described by Kim et al. [16]. Briefly, chicken breast meat (0.5 g) was placed in a tube (Millipore Ultrafree-MC, Millipore, Bedford, MA, USA) and heated in a water bath at 80 °C. After 20 min, the tube containing the samples was cooled to 23 °C and then centrifuged for 20 min at 4 °C (2000× *g*). The final WHC was calculated as follows:

WHC (%) = (moisture content − water loss)/moisture content × 100

Water loss = (weight before centrifugation − weight after centrifugation)/(sample weight × fat factor) × 100, fat factor = 1 − (crude fat%/100).

The chicken thigh meat was placed in a polyethylene bag and heated in a water bath (75 °C) for 45 min. The samples were cut into 1 × 3 × 2 cm pieces, and their shear force values were measured using a TA1 texture analyzer (Lloyd Instruments, Berwyn, IL, USA) with a V blade. The load cell and crosshead speed were 500 N and 50 mm/min, respectively.

### 2.4. Collagen Content

Collagen content was determined by measuring hydroxyproline content according to the method described by Kim et al. [15]. Briefly, each BGE sample (5 g) was hydrolyzed using 30 mL of 7 N sulfuric acid for 16 h at 105 °C. Next, 1 mL of the acid hydrolyzed-diluted sample was mixed with 0.5 mL of 1.41% chloramine T in a collagen buffer solution (pH 6.0) containing sodium hydroxide (15 g), sodium acetate trihydrate (90 g), citric acid monohydrate (30 g), and 1-propanol (290 mL) per 1 L of water. The mixture was then shaken and incubated for 20 min at 23 ± 1 °C. The mixture was then mixed with 0.5 mL of reactive color reagent (5 g of 4-dimethylaminobenzaldehyde, 17.5 mL of 60% sulfuric acid, and 32.5 mL of 2-propanol) and incubated in a water bath for 15 min at 60 °C. After the reaction was completed, the absorbance was measured at 558 nm using a UV-Vis spectrophotometer (SpectraMax M2e, Molecular Devices, Sunnyvale, CA, USA). The hydroxyproline content was calculated using a standard curve. The collagen content of the samples was calculated using a correction factor of 8.0.

### 2.5. Cholesterol Content

The cholesterol content of the chicken meat was analyzed using the Food Code [17]. Briefly, 2 g of sample (containing 5-cholestane as an internal standard) was saponified with 40 mL of 95% ethanol and 8 mL of 50% KOH at 80 °C for 70 min with a condenser. After the reactant was cooled, 60 mL of 95% ethanol was flowed through the upper part of the condenser. After hydrolysis, the reactant was extracted with n-hexane, 1 N KOH, and 0.5 N KOH, and then washed with water. The clean n-hexane layer was collected and concentrated under vacuum. The concentrated extract was dissolved in 3 mL of dimethylformamide reagent and derivatized for GC analysis (7890N, Agilent Technologies, Santa Clara, CA, USA) using an HP-5 column (30 m × 0.33 mm × 0.25 mm; Agilent Technologies). The carrier gas, flow rate, and split ratio were He (99.99%), 1.0 mL/min, and 1:12.5, respectively. The analytical temperatures of the injector and the flame ionization detector were 250 °C and 300 °C, respectively. The optimized column temperature program was as follows: the initial temperature of 190 °C was held for 2 min, and then the temperature was increased to 230 °C at a rate of 20 °C/min, held at 230 °C for 3 min, increased to 270 °C at a rate of 40 °C/min, and finally held at 270 °C for 25 min. Cholesterol content was calculated using the ratio of the target area to the internal standard area, expressed as mg/100 g of meat.

### 2.6. Nucleotide-Related Compounds

Nucleotide content was determined according to the method described by Lee et al. [18], with slight modifications. Minced samples (5 g) were mixed with 25 mL of 0.7 M perchloric acid and homogenized (Polytron R PT-2500 E, Kinematica, Luzern, Switzerland). The homogenate was centrifuged at 2000× *g* for 15 min at 0 °C and filtered through a filter paper (Whatman No. 4). The remaining pellet was re-extracted using 20 mL of 0.7 M perchloric acid and filtered through a filter paper. The collected supernatant was adjusted to pH 6.5 with 5 N KOH. The supernatant was placed in a volumetric flask, and the volume was adjusted to 100 mL with 0.7 M perchloric acid (pH 6.5, adjusted with 5 N KOH). After cooling for 30 min, the mixture was centrifuged at 1000× *g* for 10 min (0 °C). The supernatant was filtered using a 0.22-μm syringe filter and then analyzed by high-performance liquid chromatography (Agilent 1260 Infinity, Agilent Technologies) under the following analytical conditions: column, Nova-pak C18 column (150 × 3.9 mm, 4-μm particles; Waters, Milford MA, USA); eluting solution, 1% trimethylamine phosphoric acid (pH 6.5); flow rate, 1.0 mL/min; injection volume, 10 μL; running time, 30 min; column temperature, 40 °C; and detection wavelength, 254 nm. Nucleotide content was determined from a standard curve obtained using AMP, IMP, inosine, ATP, ADP, and hypoxanthine standards (Sigma Aldrich, St. Louis, MO, USA).

### 2.7. Free Amino Acid Content

The free amino acid composition of chicken thigh meat was determined as described by Lee et al. [19], with slight modifications. In brief, 2 g of chicken thigh meat were homogenized at 13,000 rpm for 30 s with 27 mL of 2% TCA solution, followed by centrifugation at 17,000× *g* for 15 min. The supernatant was filtered through a 0.45-μm syringe filter and analyzed using an amino acid analyzer (S433; SYKAM, Eresing, Germany) under the following conditions: column, 4.6 mm i.d. × 150 mm lithium-form resin; eluting solution, lithium citrate buffer (pH 2.9, 4.2, 8.0); flow rate, 0.45 mL/min (and 0.25 mL/min for ninhydrin); column temperature, 37 °C; reaction temperature, 110 °C; and analysis time, 120 min. The content of specific amino acids was determined from their respective absorption intensities, which were calibrated to known amino acid standards.

### 2.8. Fatty Acid Composition

The fatty acid composition of chicken thigh meat was analyzed as described by Kim et al. [16]. Lipids were extracted from a sample (2 g) by the addition of 40 μL of BHA and 15 mL of Folch’s solution (2:1 mixture of chloroform and methyl alcohol, *v*/*v*). The homogenates were filtered through a filter paper (Whatman No. 1). The filtrate was vortexed with 4 mL of KCl (0.88%) and centrifuged at 783× *g* for 10 min to separate the two layers. The lower lipid-containing layer was then condensed using N2. Next, 25 mg of the lipid sample was mixed with 1.5 mL of 0.5 N NaOH (in methyl alcohol) in glass tubes and heated to 100 °C for 5 min. The mixture was mixed with 1 mL of 10% BF3 and heated to 100 °C for 2 min. After the addition of 2 mL of isooctane and 1 mL of saturated NaCl, the samples were centrifuged at 783× *g* for 3 min. Iso-octane extract aliquots were injected into an Agilent 7890N gas chromatograph (Agilent Technologies) equipped with an Omegawax 250 capillary column (30 m × 0.25 mm × 0.25 mm; Supelco, Bellefonte, PA, USA). The carrier gas, flow rate, and split ratio were He (99.99%), 1.2 mL/min, and 1:100, respectively. The analytical temperatures of the injector and the flame ionization detector were 250 °C and 260 °C, respectively. The optimized column temperature program was as follows: the initial temperature of 150 °C was held for 2 min, followed by a gradual increase in temperature to 220 °C at a rate of 4 °C /min, and the temperature was finally held at 220 °C for 30 min. Each fatty acid was identified by matching its retention time with that of a respective standard, using a commercially available mixture of fatty acids (PUFA No. 2-Animal Source; Supelco).

### 2.9. Volatile Organic Compounds

The volatile organic compound (VOC) profile was determined using the headspace SPME–GC/MS analysis of Lv et al. [20]. Volatile compounds in the meat samples were isolated using the headspace solid-phase microextraction method. The fiber used for the absorption of volatiles was DVB/CAR/PDMS -50/30 μm (needle length 1 cm, needle size 24 ga) (Sigma Aldrich). Next, 5 g of the samples was homogenized in a 20-mL glass vial and incubated at 60 °C for 25 min. The fiber was then exposed to the headspace for 30 min under the same conditions. Before each analysis, the fiber was exposed to the injection port for 30 min to remove volatile contaminants. 

GC/MS analysis was performed using an Agilent 8890 gas chromatograph coupled to an Agilent 5977 B mass spectrometer (Agilent Technologies). Helium was used as the carrier gas at a constant flow rate of 1.3 mL/min. The injector was operated in the spitless mode for 5 min at 250 °C. Separation of compounds was performed on a DB-5MS column (30 m, 0.25 mm i.d., 0.25 μm film thickness; Agilent Technologies). The oven temperature was maintained at 40 °C for 5 min, programmed at 5 °C/min up to 250 °C, and held for 5 min. The interface temperature was set to 280 °C. The mass spectrometer was operated in the electron impact mode with an electron energy of 70 eV and a scan range of 30–300 m/z (scan rate, 4.37 scans/s; gain factor, 1; resulting EM voltage, 1140 V). The temperatures of the MS source and quadrupole were set to 230 °C and 150 °C, respectively. Compounds were identified by comparing the linear retention indices based on a homologous series of even numbered n-alkanes (C8-C24; Niles, IL, USA) with those of standard compounds and with literature data. Moreover, the MS data were compared with those of the reference compounds and with MS data obtained from the NIST 20 library (NIST/EPA/NIH Mass Spectral Library with Search Program) for the deconvolution of mass spectra and identification of target components. Values are expressed as the sum of the abundances of characteristic anions for each component (area × 10^6^). The flavor characteristics of the volatile compounds were searched using the following databases: Flavor DB (https://cosylab.iiitd.edu.in/flavordb/ accessed on 12 January 2022), FooDB (https://foodb.ca/ accessed on 12 January 2022), and Flavornet (http://www.flavornet.org/ accessed on 12 January 2022).

### 2.10. Sensory Characteristics

The sensory characteristics of the chicken thigh meat were evaluated by 15 panelists consisting of college students (age 21–38 years). Chicken thigh meat was served in pieces 1 × 1 × 2 cm in size. Color, aroma, flavor (1 = very bad, 9 = very good), juiciness (1 = very dry, 9 = very juicy), tenderness (1 = very tough, 9 = very tender), and texture (1 = very hard, 9 = very soft) were evaluated according to a 9-point hedonic scale. This study was approved by the Institutional Review Board (IRB) of Kangwon National University (KWNUIRB-2021-05-004-001).

### 2.11. Statistical Analysis

All analyses were performed with *n* = 10, and the data are expressed as means with standard error. Statistical analysis was performed using the SAS software (version 9.4, SAS Institute Inc., Cary, NC, USA) using one-way analysis of variance and Tukey’s test. Differences in means were considered significant at *p* < 0.05. Capital letters indicate significant differences between fresh and frozen-thawed meat within the same breed. Small letters indicate significant differences in fresh or frozen-thawed meat among different breeds.

## 3. Results and Discussion

### 3.1. Proximate Composition

The proximate composition of the chicken thigh meat was slightly influenced by both the chicken breed and the fresh or frozen-thawed state. As shown in Table 1, moisture content was the only variable affected by both factors. The fresh chicken thigh, regardless of the breed except for CB, had higher (*p* < 0.05) moisture content than frozen-thawed chicken thigh. As explained by Jeong et al. [21], the thawing of ice crystals into water causes the exudation of intracellular water, leading to increased water activity on the meat surface, which may promote moisture loss in frozen-thawed meat. The results of this study are in agreement with those of a previous report by Oliveira et al. [22]. Concerning the chicken breed, fresh thigh meat obtained from HH3 had the highest (*p* < 0.05) moisture content among all groups except for WRMD 2 (*p* > 0.05). WRMD 1 had the lowest moisture content. Similar findings were also recorded in the frozen-thawed condition, where the moisture content of WRMD 1 meat was lower (*p* < 0.05) than that of CB meat, but did not differ from that of the meat from the other KNC breeds (*p* > 0.05). Furthermore, the crude protein content was higher (*p* < 0.05) in frozen-thawed thigh meat from all KNC breeds than in CB meat. The crude fat content of fresh WRMD 2 meat was notably lower (*p* < 0.05) than that of fresh CB and HH3 meat, but not different (*p* > 0.05) from that of fresh WRMD 1 meat. Crude ash content was the lowest in fresh and frozen-thawed WRMD 2 meat. Macronutrient content is highly influenced by breed, and reportedly, macronutrient content in KNC meat is considerably higher than that in CB meat [23]. Accordingly, crude fat and crude protein content was highly influenced by the KNC breed.

### 3.2. Physicochemical Characteristics

Table 2 presents the physicochemical properties of fresh and frozen-thawed KNC and CB meat. The pH value of thigh meat from various chicken breeds under any condition in this study ranged from 6.29–6.65, which is within the standard range determined by previous studies [24,25]. The pH value was influenced (*p* < 0.05) by both the chicken breed and the fresh or frozen-thawed state. We observed notable differences in pH value in fresh thigh meat, and WRMD 2 had the lowest pH value among the different breeds (*p* < 0.05). However, no differences were observed between CB, HH3, and WRMD 1. In addition, irrespective of the chicken breed, pH value was higher (*p* < 0.05) in fresh chicken thigh than in frozen-thawed-state chicken thigh. The decrease in pH value in frozen-thawed meat might be attributed to the isoelectric alteration following the exudation of small proteins and minerals, as well as the denaturation of proteins [26]. This phenomenon has been observed by many studies [7,9,26] to also influence the WHC percentage of meat, where a lower WHC percentage is considered to highly correlate to the extent of pH decline postmortem. A decrease in the net charge of myofibrillar protein from the continuous reduction in pH close to the isoelectric point of myofibrillar proteins resulted in reduced WHC percentage. In addition, the rate of postmortem pH decline is highly dependent on lactic acid concentration owing to the anaerobic glycolytic process. A previous study reported that the pH values of any meat cut from various KNC breeds were similar to or lower than those of CB meat [24,27], and the pH value of thigh meat from KNC was similar to that of CB thigh meat. 

The surface color of meat is the most essential parameter influencing the initial purchasing intention of consumers during retail display [28]. It is highly correlated with the fresh or frozen-thawed state of meat and is influenced by factors including age, pH, breed, and sex [9]. Biochemically, it is strongly determined by the postmortem myoglobin profiles of meat, which may differ between red and white meat [29]. The lightness (CIE L*), redness (CIE a*), and yellowness (CIE b*) of chicken thigh were measured under both fresh and frozen-thawed conditions. CIE L* and CIE a* were influenced (*p* < 0.05) by either the chicken breed or fresh/frozen-thawed state. Under fresh conditions, CIE L* was the highest in HH3, followed by WRMD 2, WRMD 1, and CB. No marked differences (*p* > 0.05) in CIE L* were observed between WRMD 1 and CB. In contrast, HH3 and CB exhibited the lowest red color score, whereas WRMD 1 maintained the highest redness score. The CIE a* score of WRMD 2 was higher (*p* < 0.05) than that of CB and HH3, but was still lower than that of WRMD 1. Moreover, the CIE b* score of WRMD 1 was higher (*p* < 0.05) than that of other breeds, either fresh or frozen-thawed. In contrast, under frozen-thawed conditions, CIE L* was the highest in CB, followed by WRMD2, which showed a similar value to HH3 and WRMD1. frozen-thawed WRMD 2 and HH3 meats shared similar lightness scores, which were higher (*p* < 0.05) than that of WRMD 1 meat. Conversely, WRMD 1 maintained the highest redness score, followed by WRMD 2, which showed a similar score to HH3 and CB. All frozen-thawed KNC meats in this study displayed a higher (*p* < 0.05) CIE a* score than CB meat. A similar finding was also observed in CIE b*, with the following order from the highest to lowest scores: WRMD 1, WRMD 2, HH3, and CB (*p* < 0.05). The frozen-thawed thigh meat from CB and HH3 maintained a notably more intense red color than the fresh counterpart. No differences in red color score were observed in either WRMD 1 or WRMD 2 meat under fresh conditions (*p* > 0.05). The frozen–thawing process influences heme pigment homeostasis, wherein myoglobin may be exudated, thus accelerating myoglobin oxidation and consequently altering the color of meat to dull brown [9,11]. 

WHC reflects the ability of muscle to retain water during storage and processing, affecting moisture, thawing, and cooking loss [30,31,32]. Under fresh conditions, regardless of the chicken breed, the WHC of thigh meat from KNC was lower (*p* < 0.05) than that of CB meat. In contrast, under frozen-thawed conditions, WRMD 2 displayed the lowest WHC value among the KNC breeds (*p* < 0.05). In addition, fresh chicken thigh exhibited a considerably higher (*p* < 0.05) WHC percentage than frozen-thawed meat, regardless of the chicken breed. Leygonie et al. [11] reported that the formation of ice crystals during the freezing process was the main reason for the decline in WHC percentage. The penetration of ice crystals into the intracellular environment of the muscle damages the cell membrane, causing moisture and fluid loss during the thawing process. 

The mean value of meat shear force under freeze–thaw conditions was strongly influenced by the chicken breed. frozen-thawed thigh meat from any KNC breed showed a higher (*p* < 0.05) shear force value (22.05–23.30 N) than that CB meat (17.26 N), indicating a tougher texture. Additionally, a difference (*p* < 0.05) between the fresh and frozen-thawed state was only observed in thigh meat from CB: frozen-thawed thigh CB meat maintained a lower (*p* < 0.05) shear force value than fresh CB meat. Interestingly, the shear force of thigh meat from all KNC breeds under fresh conditions did not change after freezing and thawing. 

All KNC breeds had higher (*p* < 0.05) collagen concentrations than CB under fresh conditions. Different slaughtering ages have a strong relationship with the formation of collagen [33]. The average slaughter age of KNC is 12–13 weeks, allowing increased formation of collagen compared to the average slaughter age of CB (5 weeks) [24]. Increased collagen concentration and actin–myosin crosslinks are major contributors to increased shear force [34]. The lower collagen content in CB meat resulted in a higher tenderness level compared with any of the KNC breeds, which was in agreement with previous reports [25,27]. No difference (*p* > 0.05) in cholesterol content was observed in any chicken breed, either fresh or frozen-thawed.

### 3.3. Taste-Related Nucleotides

The taste-related nucleotides, including hypoxanthine, 5’-IMP, inosine, 5’-AMP, 5’-ADP, and 5’-ATP, along with the K value, are presented in Table 3. Under fresh conditions, hypoxanthine concentrations were higher (*p* < 0.05) in HH3 and WRMD2 than in WRMD1 and CB. Under frozen-thawed conditions, HH3 and WRMD 1 displayed higher (*p* < 0.05) hypoxanthine concentrations than CB and WRMD 2. The results of this study were slightly different from those of a previous report, which showed no differences in hypoxanthine levels between frozen-thawed CB and KNC meats [23]. According to a previous study by Jayasena et al. [24], the formation of hypoxanthine is dependent on the reserve of IMP in meat. The IMP is converted into inosine and hypoxanthine with the help of enzymes.

Therefore, this study suggested that the differences in hypoxanthine content might be caused by breed-related factors, and the use of different newly developed KNC breeds (HH3, WRMD 1, and WRMD 2) might be responsible for these differences.

5’-IMP is considered a substantial flavor precursor in meat proteins [35]. In this study, 5’-IMP concentration in both fresh and frozen-thawed chicken thigh meat was strongly dictated by the chicken breed, where both WRMD 1 and WRMD 2 maintained a similar result to that of CB. In the fresh state, no difference (*p* < 0.05) in 5’-IMP content was observed between HH3, WRMD 1, and WRMD 2. HH3 exhibited a lower (*p* > 0.05) 5’-IMP concentration than CB. Under frozen-thawed conditions, however, chicken thighs derived from HH3 had the lowest 5’-IMP concentration, whereas 5’-IMP concentrations in WRMD 1 and WRMD 2 did not differ from that in CB. These findings revealed that thigh meat from WRMD 2 may possess a desirable flavor similar to that of WRMD 1, the breed widely recognized for its tasty meat. 

In the fresh and frozen-thawed states, inosine concentration was lower (*p* < 0.05) in KNC than in CB, regardless of the KNC breed. This result was in accordance with a previous report of lower inosine content in the thigh meat of KNC than in CB [24,36]. In addition, under both fresh and frozen-thawed conditions, HH3 maintained a higher K value (*p* < 0.05) than WRMD 1. Under frozen-thawed conditions, the highest K value was observed in the thighs of HH3 chickens. No further differences in the K value were observed between CB, WRMD 1, and WRMD 2. The K value measures the degree of freshness of muscle proteins. It is a spoilage assessment marker based on the concentration of byproducts of ATP breakdown [37]. The K value of chicken thigh meat under any conditions in this study was lower than 60%, and thus fell within the edible range, as meat with a K value higher than 60% is categorized as putrefied or inedible [38]. Apart from muscle type, environmental stress, and animal genetics, fluoride loss along with protein denaturation rate are major factors that increase K value [39]. Therefore, the higher K value of frozen-thawed meat in comparison to that of fresh meat might be attributed to these causes.

### 3.4. Free Amino Acid Content

Using an amino acid analyzer, we identified 19 FAAs in the samples, as presented in Table 4. Both chicken breed and freshness state influenced the total FAA concentration owing to distinct differences in each individual FAA. These FAAs in chicken meat affect taste perception differently: Glu and Asp are umami FAAs; Thr, Ser, Gly, and Ala are sweet FAAs; Val, Met, Ile, Leu, Phe, Tyr, His, Arg, and Lys are bitter FAAs; and Asn, Trp, and Cys do not impart a specific taste perception [40]. In this study, under fresh conditions, the total FAA content was higher in CB and WRMD 1 than in WRMD2 and HH3 (*p* < 0.05). 

Under fresh conditions, the content of glutamic acid, the main umami FAA, did not differ between CB, WRMD1, and WRMD 2, but was lower (*p* < 0.05) in HH3. However, the concentration of Asp, the secondary umami FAA, was higher (*p* < 0.05) in CB than in all KNC breeds. In addition, under fresh conditions, the concentration of total sweet FAAs in CB and WRMD 1 meats was higher than that in WRMD 2 and HH3 meats. Among frozen-thawed meats, the concentration of total sweet FAAs was higher in CB meat than in HH3, WRMD 1, and WRMD 2 meats. Similarly, under fresh conditions, CB, WRMD 1, and WRMD 2 meats had a higher (*p* < 0.05) concentration of total bitter FAAs than HH3 meat. However, under frozen-thawed conditions, total bitter FAA concentration was higher (*p* < 0.05) in CB than in all KNC breeds. The abundant content of FAA inside the bone marrow were reported to shift into the meat over two weeks of frozen storage. The water-soluble compounds, including FAAs, move steadily with the influence of structural alteration due to the formation of ice crystals, creating routes for substance transmission, and causing different osmotic pressure [13]. In addition, unlike the minerals that declined over the storage period, the FAA concentration was intensified by enzymatic hydrolysis. Therefore, this study suggested that the differences in the enzymatic mechanism among chickens might create differences in FAA profiles after frozen. 

As explained by Tang et al. [41], the proportion of FAAs is highly influenced by the chicken breed and slaughtering age. Slaughtering age has a positive correlation with the formation of umami-related non-volatile compounds, including Glu, due to increased protein percentage, whereas chicken breed determines the FAA composition of different muscle fiber types. Interestingly, in this study, we found that the concentration of each individual FAA, except for taurine, tryptophan, and carnosine, was higher in frozen-thawed meat than in fresh meat, irrespective of the chicken breed. Two main factors may be responsible for the increased concentration of FAA in chicken meat following freezing and thawing: (1) the structural damage of muscle tissue due to a formation of ice crystals during freezing, which promotes the migration of FAA from the bone marrow into muscle tissue; and (2) hydrolysis of protease [13]. 

### 3.5. Fatty Acid Composition

The fatty acid composition profiles of the KNC breeds are presented in Table 5. Based on comparison of the mean value, the individual fatty acid composition of chicken thigh meat was influenced by both the chicken breed and the fresh or frozen-thawed state. The saturated fatty acid (SFA), monounsaturated fatty acid (MUFA), and polyunsaturated fatty acid (PUFA) profiles strongly differed according to the chicken breed. Under fresh conditions, the percentage of PUFAs was the highest in HH3 and WRMD 1, followed by WRMD 2. In contrast, all KNC breeds in this study showed higher (*p* < 0.05) PUFA proportion than CB. This finding is in agreement with the results of Lee et al. [7] and Jayasena et al. [24], in which the PUFA content in KNC meat was notably higher than that in CB meat. In contrast, the concentrations of MUFAs were higher (*p* < 0.05) in CB than in the KNC breeds. Furthermore, under fresh conditions, WRMD 2 and CB showed the highest (*p* < 0.05) SFA composition, followed by HH3 and WRMD 1. The SFAs myristic acid and stearic acid were the main contributors to the high SFA percentage in WRMD 2.

In this study, the predominant fatty acids were oleic, palmitic, linoleic, and stearic acids. Moreover, the proportions of the essential fatty acids linoleic and arachidonic acid were higher (*p* < 0.05) in all KNC breeds than in CB under both fresh and frozen-thawed conditions. This indicated that the newly developed KNC breeds, WRMD 2, provided similar advantages to previously recognized KNC breeds (HH3 and WRMD 1). In addition, the higher content of essential fatty acids in KNC is in agreement with previous reports [7,24,36]. Furthermore, certain fatty acids are closely related to taste perception. Docosahexaenoic acid is characterized by its sweet and bitter taste; arachidonic acid is closely correlated with an umami taste; and oleic and linoleic acids contribute to a salty and sour taste, respectively [6,25,42]. Under fresh and frozen-thawed conditions, DHA concentration was the highest in WRMD 2, followed by HH3, WRMD 1, and CB (*p* < 0.05). The percentage of DHA was the lowest in both fresh and frozen-thawed CB meat. Regardless of the KNC breed and fresh or frozen-thawed conditions, the content of arachidonic acid, an umami fatty acid, was higher (*p* < 0.05) in KNC than in CB. However, the percentage of oleic acid in thigh meat from all KNC breeds was lower than that in CB. These findings on fatty acid composition suggest that the newly developed KNC breed, WRMD 2, can provide both essential nutrients and a favorable taste.

### 3.6. Volatile Organic Compounds

Flavor is composed of a combination of taste and aroma, and it is an essential factor affecting the repurchasing intention of consumers toward meat products [43]. In general, perceived flavor and aroma are strongly determined by both the individual VOC and the VOC class [44]. Although characterization studies on the flavor profiles of KNC have been conducted, an in-depth study of the newly developed breed WRMD 2 has not been carried out. As shown in Table 6, 142 VOCs were obtained, grouped into hydrocarbons (57), esters (17), alcohols (22), aldehydes (20), acids (10), ketones (8), and other compounds (8). Higher amounts of total alcohol, aldehyde, ketones, and other compounds were observed in frozen-thawed CB meat than in fresh CB meat (*p* < 0.05). Conversely, the number of VOCs, especially total hydrocarbons, aldehydes, acids, ketones, and other compounds, was higher (*p* < 0.05) in fresh WRMD 1 meat than in frozen-thawed WRMD 1 meat. Additionally, the amount of total ester was higher (*p* < 0.05) in fresh meat than in frozen-thawed meat, irrespective of the chicken breed. Among the chicken breeds, WRMD 1 showed the highest content of ketones under fresh conditions. In contrast, under frozen-thawed conditions, the total amounts of alcohols, aldehydes, and ketones were higher in CB than in the KNC breeds (*p* < 0.05). Similarly to these findings, the predominant VOCs in chicken meat are hydrocarbons [45].

Studies have reported aldehydes as the most essential flavoring substances owing to their low odor threshold [13,46]. This compound is the main product of the lipolysis of fatty acids, primarily PUFAs. It contains a highly vulnerable bond, making it less stable than SFAs and MUFAs [13,47]. Furthermore, among aldehydes in meat proteins that induce sensory perceptions, nonanal and octanal are categorized as pleasant compounds, whereas pentanal and hexanal are unpleasant compounds [48]. Pleasant volatile compounds, including nonanal (rose, orange, meaty), octanal (lemon, citrus, soap, orange peel, fat, and fruity), 2-methyl butanal (chocolate, cocoa, mocha, coffee, almond), 3-methyl butanal (malt, almond, chocolate), 2-methyl propanal (aldehydic, pungent, floral), and benzaldehyde (almond, burnt sugar), have been reported to be decreased or even lost during cold storage owing to excessive lipid oxidation in chicken meat [49]. Interestingly, the present study showed a different trend in aldehyde concentrations between CB and KNC after freezing. The concentrations of 2-nonenal, (E)- (aldehydic, citrus, cucumber, fat), 2-octenal, (E)- (green, nut, fat), decanal (soap, orange peel, tallow), 2,4-decadienal, (E,E) (Asian pear, asparagus, corn, orange mint), nonanal, and octanal aldehydes increased remarkably in CB, but decreased in KNC breeds. Qi et al. [13] reported that the increase in pleasant VOC content was due to the higher exposure of hydrophobic compounds, whereas the decreased pleasant VOC content was mainly due to lipid oxidation at −18 °C [50]. Numerous factors are strongly correlated with the flavor development of chicken meat; for instance, age, breed, sex, diet, age at slaughter, and storage conditions [51,52]. Different animal breeds produce different organoleptic perception and palatability [53]. Moreover, the high contents of bioactive and taste-active compounds in KNC generate an intense and unique flavor that is preferable for consumers [24,54].

In addition, the results of this study revealed different trends of changes in other VOC contents after freezing between CB and KNC. The concentrations of ketones, 2-pentylfuran (green bean, butter), and octanoic acid (cheesy, sweat, vegetable, waxy, fatty) were higher in fresh CB and lower in frozen-thawed KNC meat (*p* < 0.05). Nevertheless, despite these differences, the freezing process affected VOC content in frozen-thawed chicken thigh meat, regardless of the chicken breed. The hydrocarbons D-limonene (mint, lemon, citrus, orange, fresh, sweet), heptadecane (alkane), hexadecane (alkane), naphthalene (dry, pungent, tarry, tar), pentadecane, 3-methyl- (alkane), and tridecane (alkane); the esters arsenous acid, tris(trimethylsilyl) ester (odorless), decanoic acid, ethyl ester (apple, brandy, waxy, grape, oily, sweet, fruity, pear), hexadecanoic acid, and ethyl ester (Asian pear, blackberry, breakfast cereal, coriander); the ketones 5,9-undecadien-2-one and 6,10-dimethyl- (odorless); the alcohols 1-hexanol and 2-ethyl-alcohol (odorless); and the aldehydes pentadecanal (fresh, waxy), tetradecanal (citrus peel, incense, amber, waxy, fatty), and tridecanal (grapefruit peel, citrus, must, fresh, waxy, sweet) aldehydes were present in lower (*p* < 0.05) concentrations in frozen-thawed meat than in fresh meat. The results were in line with those of Qi et al. [13], who mentioned that as the development of VOCs are mainly due to PUFAs, which have a lower rate of stability compared to both MUFAs and SFAs, lipolysis of phospholipids occurs during the frozen storage of meat, and is assumed to be the main factor for the intensification of aroma-active compounds, which was also observed in this study. 

However, regardless of the chicken breed, the contents of certain VOCs were decreased or even lost in frozen-thawed meat. Pentadecane (alkane), tetradecane (alkane, mild, waxy), dodecane, 2,6,11-trimethyl- (alkane), 4,6-dimethyl- (alkane), n-hexane (alkane), and 2,4-dimethyldodecane (odorless) hydrocarbons were lost in frozen-thawed meat, and the concentrations of n-hexane, benzene, and 1,3-bis(1,1-dimethylethyl)- were lower (*p* < 0.05) in frozen-thawed meat from all KNC breeds, especially HH3 and WRMD2. Butylated hydroxytoluene (odorless) ester, 1-hexanol, and 2-ethyl-alcohol (odorless) were also affected by the freezing process, and their concentrations were decreased (*p* < 0.05) in frozen-thawed thigh meat from KNC breeds. Qi et al. [13] revealed lipid degradation as a main factor in the development of VOCs, and heating and storage are believed to be important contributors to this process. Most VOCs in meat are derived from PUFAs, particularly oleic and linoleic acids. During oxidation, the unsaturated bonds of PUFAs, which are vulnerable under stress conditions, will induce the formation of most major VOCs, such as octanal, hexanal, heptanal, and nonanal [55]. Hexanal may further react to generate 4,5-dimethyl-2-pentyl-3-oxazoline, which produces an unfavorable aroma perception, whereas nonanal contributes to the meaty aroma when converted into 12-methyltridecanal [56]. These VOCs are presumed to be markers of lipid oxidation during the dry heating of red meat [57]. Furthermore, considering the results of this VOC analysis, we assume that the changes in diverse VOCs of the hydrocarbon (d-limonene, heptadecane, hexadecane, naphthalene, pentadecane, 3-methyl-, tridecane), ester (arsenous acid, tris(trimethylsilyl) ester, decanoic acid, ethyl ester, hexadecanoic acid, ethyl ester), alcohol (1-hexanol, 2-ethyl-), ketone (5,9-undecadien-2-one, 6,10-dimethyl-), and aldehyde (pentadecanal-, tetradecanal, tridecanal) classes can be prominent marker compounds for distinguishing between fresh and frozen-thawed chicken thigh meat.

### 3.7. Sensory Evaluation

The sensory characteristics of thigh meat from each chicken breed are presented in Table 7. Under fresh conditions, the flavor profile and juiciness level were affected (*p* < 0.05) by the chicken breed, with WRMD 2 showing a lower (*p* < 0.05) flavor score than CB and WRMD 1. However, the score for flavor perception did not differ (*p* > 0.05) from that of HH3. In addition, juiciness score was the highest in HH3, and no differences (*p* > 0.05) were observed between CB, WRMD 1, and WRMD 2. Under frozen-thawed conditions, however, tenderness score in HH3 was lower than that in CB, while this flavor perception was similar between HH3 and the other KNC breeds (WRMD 1 and WRMD 2). The higher collagen content may have been responsible for the lower perception of tenderness by the panelists, as collagen content influences the texture of chicken meat [51]. Additionally, the color score of frozen-thawed meat was lower in WRMD 1 than in CB. However, the score for color perception of WRMD 1 did not differ from that of the other KNC breeds (HH3 and WRMD 2). Freshness particularly affected the organoleptic perception of WRMD 2 meat, wherein panelists gave a higher (*p* < 0.05) score for frozen-thawed meat with respect to taste, flavor, and overall acceptability. Similarly, for CB, the tenderness score was higher for the frozen-thawed meat than for the fresh meat. The results of the sensory evaluation contrasted with those reported by Bae et al. [9] and Leygonie et al. [11], wherein the thawing process promoted fluid and moisture loss in frozen-thawed meat owing to the shrinkage of muscle fibers, resulting in a lower sensory score. The slightly different trend in this study might be due to the difficulty for untrained panelists in clearly distinguishing each sensory attribute, especially taste and flavor. As reported by Qi et al. [13], the ability of panelists to distinctly recognize various samples is highly determined by the number of trainings undergone through exposure to reference samples. Therefore, further studies should be performed to confirm the results of the current study. No further differences (*p* > 0.05) were observed in the perceived texture and overall acceptability between chicken breeds or fresh and frozen-thawed meats.

## 4. Conclusions

The physicochemical characteristics and organoleptic attributes of fresh and frozen-thawed chicken thigh meat from different breeds were compared. The newly developed KNC breeds WRMD 1 and WRMD 2 exhibited similar taste-related nucleotides, pleasant and essential fatty acids, and flavor profiles to HH3, which was previously recognized as a premium breed in Korea. Although higher in collagen content, KNC breeds showed no significant differences in shear force value when compared to CB, with the same result for overall acceptability in the sensory evaluation test. Freezing intensified the flavor-active compounds, including nucleotides, FAA, and VOCs in chickens; however, it caused the depletion of favorable VOCs in WRMD1. The changes in VOC clusters, including some hydrocarbons, esters, alcohols, ketones, and aldehydes, are suggested to be a prominent marker in distinguishing between fresh and frozen-thawed chicken meat. Further studies to determine other taste-active compounds, such as dipeptides, free amino acids, and volatile compounds, are required to gain a deeper understanding of the organoleptic compounds of chicken meat from various breeds when processed under any given conditions.

## Figures and Tables

**Table 1:** Comparison of proximate composition of fresh and frozen-thawed chicken meats from Korean native chickens and broilers.

Proximate Composition (%)	Broiler		HH3		WRMD1		WRMD2	
	Fresh	Frozen-Thawed	Fresh	Frozen-Thawed	Fresh	Frozen-Thawed	Fresh	Frozen-Thawed
Moisture	76.11 ± 0.48 ^b^	76.03 ± 2.60 ^a^	77.14 ± 0.70 ^Aa^	74.68 ± 0.76 ^Bab^	74.93 ± 0.58 ^Ac^	72.14 ± 1.14 ^Bb^	76.95 ± 0.44 ^Aab^	72.94 ± 1.90 ^Bab^
Crude protein	18.89 ± 0.97	18.61 ± 0.33 ^b^	19.55 ± 0.61	20.05 ± 0.72 ^a^	20.15 ± 1.21	20.39 ± 0.74 ^a^	19.44 ± 0.56	20.05 ± 0.50 ^a^
Crude fat	5.71 ± 0.58 ^a^	6.02 ± 1.03	4.32 ± 0.40 ^a^	5.14 ± 1.33	4.91 ± 0.82 ^ab^	6.20 ± 1.24	4.07 ± 0.70 ^b^	4.62 ± 0.54
Crude ash	1.10 ± 0.24	1.17 ± 0.22 ^ab^	1.04 ± 0.20	1.09 ± 0.26 ^a^	1.10 ± 0.21	0.99 ± 0.12 ^ab^	0.92 ± 0.25	0.77 ± 0.15 ^b^

HH3, Hanhyup No.3; WRMD1, Woorimatdag No.1; WRMD2, Woorimatdag No.2. ^A,B^ Different letters represent a significant difference between fresh and frozen-thawed meat within the same breed (*p* < 0.05). ^a–c^ Different letters represent a significant difference between the fresh or frozen-thawed meat of different chicken breeds (*p* < 0.05). Mean ± SD.

**Table 2:** Comparison of quality properties of fresh and frozen-thawed chicken meats from Korean native chickens and broilers.

Variables	Broiler		HH3		WRMD1		WRMD2	
	Fresh	Frozen-Thawed	Fresh	Frozen-Thawed	Fresh	Frozen-Thawed	Fresh	Frozen-Thawed
pH	6.65 ± 0.08 ^Aa^	6.47 ± 0.07 ^B^	6.63 ± 0.06 ^Aa^	6.48 ± 0.02 ^B^	6.63 ± 0.09 ^Aa^	6.38 ± 0.02 ^B^	6.44 ± 0.06 ^Ab^	6.29 ± 0.05 ^B^
CIE L*	47.01 ± 1.34 ^Bc^	54.42 ± 1.33 ^Aa^	56.27 ± 0.57 ^Aa^	50.34 ± 1.25 ^Bb^	47.46 ± 2.17 ^Ac^	44.99 ± 0.67 ^Bc^	49.92 ± 0.62 ^Bb^	51.66 ± 0.93 ^Ab^
CIE a*	4.06 ± 0.52 ^Bc^	6.37 ± 0.65 ^Ac^	3.36 ± 0.36 ^Bc^	8.88 ± 0.67 ^Ab^	12.23 ± 0.53 ^a^	12.76 ± 0.70 ^a^	9.81 ± 0.59 ^b^	9.70 ± 1.26 ^b^
CIE b*	8.08 ± 0.52 ^b^	8.21 ± 0.55 ^c^	7.10 ± 0.24 ^Bbc^	9.94 ± 0.35 ^Ab^	9.72 ± 0.83 ^Ba^	11.86 ± 1.20 ^Aa^	6.87 ± 0.52 ^Bc^	10.22 ± 0.34 ^Ab^
WHC (%)	84.51 ± 0.48 ^Aa^	60.77 ± 0.48 ^Ba^	79.79 ± 0.70 ^Ab^	58.31 ± 0.70 ^Bab^	80.22 ± 0.58 ^Ab^	59.93 ± 0.58 ^Ba^	80.24 ± 0.44 ^Ab^	52.34 ± 0.44 ^Bb^
Shear force (N)	26.31 ± 3.06 ^A^	17.26 ± 3.06 ^Bb^	24.37 ± 1.83	23.30 ± 1.83 ^a^	24.71 ± 3.11	23.19 ± 3.11 ^a^	24.01 ± 3.70	22.05 ± 3.70 ^a^
Collagen contents (mg/100 g)	1.14 ± 0.08 ^b^	1.14 ± 0.09 ^b^	1.37 ± 0.11 ^a^	1.36 ± 0.12 ^ab^	1.42 ± 0.16 ^a^	1.39 ± 0.17 ^a^	1.40 ± 0.08 ^a^	1.35 ± 0.12 ^ab^
Cholesterol (mg/100 g)	91.80 ± 9.23	91.75 ± 2.44	84.83 ± 9.63	89.64 ± 8.04	91.17 ± 7.32	99.24 ± 8.03	78.38 ± 7.64	89.35 ± 13.49

HH3, Hanhyup No.3; WRMD1, Woorimatdag No.1; WRMD2, Woorimatdag No.2. WHC; Water holding capacity. ^A,B^ Different letters represent a significant difference between fresh and frozen-thawed meat within the same breed (*p* < 0.05). ^a–c^ Different letters represent a significant difference between the fresh or frozen-thawed meat of different chicken breeds (*p* < 0.05). Mean ± SD.

**Table 3:** Comparison of nucleotide-related compounds contents of fresh and frozen-thawed chicken meats from Korean native chickens and broilers.

Nucleotide-Related Compounds (mg/100 g)	Broiler		HH3		WRMD1		WRMD2	
	Fresh	Frozen-Thawed	Fresh	Frozen-Thawed	Fresh	Frozen-Thawed	Fresh	Frozen-Thawed
Hypoxanthine	31.79 ± 2.90 ^c^	33.84 ± 2.08 ^b^	40.05 ± 1.90 ^a^	42.20 ± 2.23 ^a^	33.29 ± 3.92 ^Bbc^	43.39 ± 1.94 ^Aa^	38.16 ± 3.57 ^ab^	36.09 ± 3.04 ^b^
IMP	97.30 ± 12.03 ^Aa^	60.09 ± 5.59 ^Ba^	72.46 ± 10.62 ^Ab^	38.25 ± 3.17 ^Bb^	93.38 ± 11.62 ^Aab^	61.92 ± 3.73 ^Ba^	89.76 ± 13.16 ^Aab^	54.05 ± 8.45 ^Ba^
Inosine	43.56 ± 6.85 ^a^	45.42 ± 3.89 ^a^	30.59 ± 2.36 ^b^	31.83 ± 0.99 ^bc^	29.17 ± 7.62 ^b^	27.00 ± 1.61 ^c^	33.8 ± 2.13 ^b^	37.04 ± 3.93 ^b^
AMP	6.78 ± 0.57 ^Bab^	7.66 ± 0.59 ^A^	6.69 ± 0.33 ^ab^	7.44 ± 1.37	5.41 ± 1.43 ^b^	7.05 ± 1.08	7.03 ± 0.62 ^a^	7.68 ± 0.24
ADP	7.17 ± 1.48 ^A^	5.55 ± 0.50 ^B^	8.41 ± 0.46 ^A^	6.54 ± 1.14 ^B^	8.98 ± 2.34	6.25 ± 1.17	7.82 ± 0.67 ^A^	6.18 ± 1.18 ^B^
ATP	7.86 ± 1.00 ^ab^	7.89 ± 0.87	9.28 ± 0.81 ^Aab^	7.25 ± 1.24 ^B^	6.38 ± 3.06 ^b^	7.86 ± 0.79	9.55 ± 0.89 ^Aa^	7.29 ± 0.40 ^B^
K value	38.82 ± 2.69 ^Bab^	49.40 ± 3.00 ^Ab^	42.29 ± 0.38 ^Ba^	55.49 ± 1.73 ^Aa^	35.53 ± 3.66 ^Bb^	45.86 ± 1.32 ^Ab^	38.82 ± 2.91 ^Bab^	49.36 ± 0.40 ^Ab^

HH3, Hanhyup No.3; WRMD1, Woorimatdag No.1; WRMD2, Woorimatdag No.2; IMP, inosine monophosphate; AMP, adenosine monophosphate; ADP, adenosine diphosphate; ATP, adenosine 5’-triphosphate. ^A,B^ Different letters represent a significant difference between fresh and frozen-thawed meat within the same breed (*p* < 0.05). ^a–c^ Different letters represent a significant difference between the fresh or frozen-thawed meat of different chicken breeds (*p* < 0.05). Mean ± SD.

**Table 4:** Comparison of free amino acids (FAA) in fresh and frozen-thawed chicken thigh meat from Korean native chickens and broilers.

FAA (mg/100 g)	Broiler		HH3		WRMD1		WRMD2	
	Fresh	Frozen-Thawed	Fresh	Frozen-Thawed	Fresh	Frozen-Thawed	Fresh	Frozen-Thawed
Taurine	47.34 ± 2.024 ^Aa^	35.13 ± 0.754 ^Bc^	41.62 ± 4.314 ^Ab^	36.50 ± 0.817 ^Bb^	40.64 ± 2.345 ^Ab^	41.31 ± 0.569 ^Aa^	39.29 ± 2.548 ^Ab^	41.99 ± 0.755 ^Aa^
Aspartic acid	14.70 ± 2.398 ^Ba^	19.46 ± 0.417 ^Aab^	5.43 ± 1.659 ^Bb^	16.40 ± 1.014 ^Ac^	5.89 ± 1.288 ^Bb^	21.70 ± 2.100 ^Aa^	6.56 ± 1.280 ^Bb^	17.02 ± 1.996 ^Abc^
Threonine	9.23 ± 1.209 ^Ba^	19.47 ± 0.905 ^Aa^	4.54 ± 1.025 ^Bc^	14.11 ± 1.215 ^Ab^	6.98 ± 1.894 ^Bab^	16.39 ± 2.571 ^Ab^	5.22 ± 1.055 ^Bbc^	14.04 ± 0.994 ^Ab^
Serine	18.03 ± 5.039 ^Ba^	35.35 ± 1.761 ^Aa^	8.33 ± 2.665 ^Bc^	21.94 ± 1.473 ^Ab^	14.31 ± 2.228 ^Bab^	23.77 ± 3.974 ^Ab^	11.10 ± 1.856 ^Bbc^	22.02 ± 2.065 ^Ab^
Asparagine	1.65 ± 0.621 ^Ba^	3.81 ± 0.097 ^Aa^	0.80 ± 0.189 ^Bb^	1.81 ± 0.196 ^Ab^	1.50 ± 0.457 ^Bab^	2.21 ± 0.426 ^Ab^	1.14 ± 0.410 ^Bab^	1.78 ± 0.242 ^Ab^
Glutamic acid	13.96 ± 3.612 ^Ba^	35.26 ± 2.927 ^Aa^	8.28 ± 2.721 ^Bb^	25.32 ± 2.466 ^Ac^	14.47 ± 3.232 ^Ba^	31.52 ± 4.130 ^Aab^	11.00 ± 1.876 ^Bab^	28.77 ± 1.827 ^Abc^
Glycine	19.40 ± 1.993 ^Ba^	38.19 ± 2.725 ^Aa^	8.16 ± 2.541 ^Bc^	26.10 ± 1.180 ^Ab^	19.51 ± 1.506 ^Ba^	25.35 ± 3.082 ^Ab^	12.96 ± 2.287 ^Bb^	22.70 ± 1.936 ^Ab^
Alanine	24.76 ± 3.512 ^Ba^	43.46 ± 1.575 ^Aa^	13.96 ± 3.623 ^Bb^	29.72 ± 1.632 ^Ab^	25.63 ± 3.387 ^Ba^	32.85 ± 3.989 ^Ab^	15.17 ± 2.391 ^Bb^	31.78 ± 2.746 ^Ab^
Valine	6.14 ± 1.468 ^Ba^	16.05 ± 0.701 ^Aa^	2.37 ± 0.727 ^Bb^	11.08 ± 1.130 ^Ab^	5.94 ± 2.467 ^Ba^	11.14 ± 2.738 ^Ab^	4.09 ± 0.866 ^Bab^	8.62 ± 1.203 ^Ab^
Methionine	2.11 ± 0.327 ^Ba^	5.67 ± 0.401 ^Aa^	0.78 ± 0.180 ^Bb^	3.56 ± 0.271 ^Ab^	2.62 ± 1.090 ^Aa^	3.25 ± 0.926 ^Ab^	1.62 ± 0.417 ^Bab^	2.83 ± 0.379 ^Ab^
Isoleucine	3.74 ± 0.838 ^Ba^	9.54 ± 0.458 ^Aa^	1.34 ± 0.352 ^Bb^	6.42 ± 0.551 ^Ab^	3.32 ± 1.369 ^Ba^	6.60 ± 1.616 ^Ab^	2.50 ± 0.565 ^Bab^	4.96 ± 0.719 ^Ab^
Leucine	6.51 ± 1.508 ^Ba^	17.90 ± 0.741 ^Aa^	2.54 ± 0.588 ^Bb^	11.44 ± 1.133 ^Ab^	6.49 ± 2.999 ^Ba^	11.97 ± 3.118 ^Ab^	4.53 ± 0.995 ^Bab^	9.05 ± 1.123 ^Ab^
Tyrosine	2.89 ± 0.648 ^Ba^	8.47 ± 0.316 ^Aa^	1.15 ± 0.343 ^Ab^	4.94 ± 0.571 ^Bb^	3.03 ± 1.297 ^Ba^	4.95 ± 1.338 ^Ab^	1.86 ± 0.465 ^Bab^	4.19 ± 0.675 ^Ab^
Phenylalanine	2.60 ± 0.680 ^Ba^	7.25 ± 0.497 ^Aa^	0.97 ± 0.240 ^Bb^	3.68 ± 1.439 ^Ab^	2.88 ± 1.177 ^Aa^	3.27 ± 0.990 ^Ab^	1.80 ± 0.384 ^Aab^	2.19 ± 0.402 ^Ab^
Histidine	2.79 ± 0.580 ^Ba^	6.35 ± 0.217 ^Aa^	0.99 ± 0.234 ^Bb^	4.61 ± 0.442 ^Ab^	2.55 ± 1.026 ^Ba^	4.64 ± 0.954 ^Ab^	1.96 ± 0.335 ^Aab^	3.91 ± 0.562 ^Ab^
Tryptophan	27.79 ± 3.559 ^Aa^	6.67 ± 0.792 ^Bc^	7.75 ± 2.296 ^Ac^	8.54 ± 0.456 ^Ab^	12.52 ± 2.661 ^Abc^	11.87 ± 0.650 ^Aa^	15.15 ± 2.377 ^Ab^	7.33 ± 0.950 ^Bbc^
Carnosine	36.19 ± 1.286 ^Aa^	9.22 ± 0.496 ^Bc^	19.51 ± 6.751 ^Ab^	13.89 ± 0.579 ^Ab^	25.92 ± 3.015 ^Ab^	16.44 ± 1.043 ^Ba^	21.93 ± 3.763 ^Ab^	16.44 ± 2.195 ^Ba^
Lysine	9.06 ± 0.770 ^Ba^	18.67 ± 1.074 ^Aa^	4.19 ± 1.499 ^Bb^	13.20 ± 2.822 ^Ab^	8.56 ± 4.834 ^Aab^	14.41 ± 3.614 ^Aab^	4.78 ± 1.145 ^Bab^	12.59 ± 1.218 ^Ab^
Arginine	5.10 ± 0.750 ^Bab^	14.62 ± 1.088 ^Aa^	2.36 ± 0.840 ^Bc^	7.94 ± 0.874 ^Ab^	6.65 ± 1.704 ^Aa^	7.94 ± 1.508 ^Ab^	3.63 ± 0.691 ^Bbc^	7.48 ± 0.792 ^Ab^
Total FAA	253.98 ± 24.491 ^Ba^	350.54 ± 12.209 ^Aa^	135.07 ± 31.828 ^Bc^	261.18 ± 15.301 ^Ab^	209.39 ± 30.992 ^Bab^	291.57 ± 37.161 ^Ab^	166.28 ± 22.960 ^Bbc^	259.71 ± 20.852 ^Ab^
Sweet FAA	71.42 ± 11.145 ^Ba^	136.47 ± 5.654 ^Aa^	34.98 ± 9.796 ^Bb^	91.87 ± 4.744 ^Ab^	66.44 ± 7.931 ^Ba^	98.36 ± 13.366 ^Ab^	44.44 ± 7.387 ^Bb^	90.54 ± 7.488 ^Ab^
Bitter FAA	21.10 ± 4.727 ^Ba^	56.41 ± 2.659 ^Aa^	8.00 ± 2.068 ^Bb^	36.18 ± 3.111 ^Ab^	21.26 ± 9.099 ^Ba^	36.22 ± 9.305 ^Ab^	14.52 ± 3.159 ^Bab^	27.66 ± 3.768 ^Ab^
Acid FAA	31.42 ± 6.239 ^Ba^	61.07 ± 3.209 ^Aa^	14.70 ± 4.582 ^Bb^	46.33 ± 3.607 ^Ac^	22.92 ± 5.360 ^Bab^	57.85 ± 7.088 ^Aab^	19.52 ± 3.350 ^Bb^	49.70 ± 3.854 ^Abc^

HH3, Hanhyup No.3; WRMD1, Woorimatdag No.1; WRMD2, Woorimatdag No.2. Bitter FAA = sum of leucine, valine, isoleucine, methionine and phenylalanine; Acid FAA = glutamic acid, aspartic acid and histidine. ^A,B^ Different letters represent a significant difference between fresh and frozen-thawed meat within the same breed (*p* < 0.05). ^a–c^ Different letters represent a significant difference between the fresh or frozen-thawed meat of different chicken breeds (*p* < 0.05). Mean ± SD.

**Table 5:** Comparison of fatty acid composition of fresh and frozen-thawed chicken thigh meat from Korean native chickens and broilers.

Fatty Acids (%)	Broiler		HH3		WRMD1		WRMD2	
	Fresh	Frozen-Thawed	Fresh	Frozen-Thawed	Fresh	Frozen-Thawed	Fresh	Frozen-Thawed
C14:0	1.07 ± 0.03 ^a^	1.07 ± 0.02 ^a^	0.92 ± 0.04 ^b^	1.06 ± 0.13 ^a^	0.79 ± 0.03 ^c^	0.81 ± 0.10 ^b^	1.02 ± 0.05 ^a^	0.98 ± 0.15 ^ab^
C16:0	24.44 ± 0.18 ^a^	24.65 ± 0.83 ^ab^	22.36 ± 0.61 ^Bc^	23.75 ± 0.85 ^A^^ab^	21.72 ± 0.64 ^c^	22.49 ± 0.70 ^b^	23.46 ± 0.41 ^b^	23.09 ± 1.20 ^ab^
C16:1 n7	6.00 ± 0.82 ^a^	6.58 ± 0.40 ^a^	4.84 ± 0.40 ^B^^b^	5.79 ± 0.76 ^A^^ab^	4.55 ± 0.63 ^b^	4.89 ± 0.85 ^b^	5.41 ± 0.42 ^ab^	5.42 ± 0.66 ^ab^
C18:0	7.50 ± 0.43 ^Ab^	6.86 ± 0.27 ^B^	7.64 ± 0.42 ^b^	6.95 ± 0.62	7.15 ± 0.29 ^b^	6.82 ± 0.52	8.79 ± 0.38 ^Aa^	7.60 ± 0.60 ^B^
C18:1 n9	41.22 ± 0.63 ^a^	41.29 ± 0.36 ^a^	35.13 ± 1.08 ^Bc^	37.37 ± 1.79 ^A^^b^	36.63 ± 0.72 ^Bb^	38.01 ± 0.83 ^A^^b^	35.41 ± 0.59 ^bc^	36.42 ± 1.16 ^b^
C18:1 n7	2.79 ± 0.15 ^a^	2.66 ± 0.11 ^ab^	2.59 ± 0.12 ^ab^	2.73 ± 0.23 ^a^	2.68 ± 0.10 ^a^	2.54 ± 0.13 ^ab^	2.42 ± 0.07 ^b^	2.39 ± 0.14 ^b^
C18:2 n6	14.06 ± 0.86 ^c^	14.43 ± 1.16 ^b^	21.01 ± 0.47 ^Aa^	18.33 ± 1.89 ^Ba^	21.54 ± 1.00 ^a^	20.69 ± 2.02 ^a^	18.39 ± 0.68 ^b^	19.72 ± 1.53 ^a^
C18:3 n6	0.13 ± 0.01 ^Bc^	0.17 ± 0.03 ^A^	0.14 ± 0.03 ^ab^	0.18 ± 0.04	0.18 ± 0.03 ^a^	0.22 ± 0.04	0.14 ± 0.02 ^Bb^	0.22 ± 0.02 ^A^
C18:3 n3	0.72 ± 0.03 ^d^	0.72 ± 0.03 ^c^	1.14 ± 0.04 ^Aa^	0.88 ± 0.09 ^Bb^	0.83 ± 0.05 ^c^	0.86 ± 0.02 ^b^	0.94 ± 0.03 ^b^	0.99 ± 0.07 ^a^
C20:1 n9	0.46 ± 0.02 ^A^	0.35 ± 0.03 ^B^^ab^	0.43 ± 0.01 ^A^	0.38 ± 0.02 ^Ba^	0.45 ± 0.03 ^A^	0.34 ± 0.04 ^Bab^	0.44 ± 0.03 ^A^	0.32 ± 0.02 ^Bb^
C20:4 n6	1.07 ± 0.08 ^Ab^	0.79 ± 0.08 ^B^^b^	2.70 ± 0.37 ^Aa^	1.85 ± 0.47 ^Ba^	2.40 ± 0.23 ^Aa^	1.69 ± 0.16 ^Ba^	2.47 ± 0.26 ^Aa^	1.98 ± 0.37 ^Ba^
C20:5 n3	0.11 ± 0.01 ^Ab^	0.06 ± 0.01 ^Ba^	0.09 ± 0.01 ^Ab^	0.04 ± 0.01 ^Bab^	0.11 ± 0.01 ^Ab^	0.03 ± 0.01 ^Bb^	0.16 ± 0.03 ^Aa^	0.06 ± 0.02 ^Ba^
C22:4 n6	0.25 ± 0.03 ^c^	0.22 ± 0.02 ^b^	0.72 ± 0.11 ^Aa^	0.52 ± 0.10 ^Ba^	0.75 ± 0.09 ^Aa^	0.48 ± 0.04 ^Ba^	0.46 ± 0.05 ^b^	0.42 ± 0.11 ^a^
C22:6 n3	0.19 ± 0.03 ^Ac^	0.15 ± 0.02 ^Bb^	0.28 ± 0.04 ^b^	0.19 ± 0.12 ^b^	0.22 ± 0.02 ^Abc^	0.14 ± 0.02 ^Bb^	0.49 ± 0.07 ^a^	0.39 ± 0.12 ^a^
SFA	33.00 ± 0.46 ^a^	32.58 ± 1.00 ^a^	30.92 ± 0.96 ^b^	31.75 ± 0.49 ^ab^	29.66 ± 0.63 ^c^	30.12 ± 1.02 ^b^	33.27 ± 0.58 ^a^	31.67 ± 1.71 ^ab^
UFA	67.00 ± 0.46 ^c^	67.42 ± 1.00 ^b^	69.08 ± 0.96 ^b^	68.25 ± 0.49 ^ab^	70.34 ± 0.63 ^a^	69.88 ± 1.02 ^a^	66.73 ± 0.58 ^c^	68.33 ± 1.71 ^ab^
MUFA	50.47 ± 1.25 ^a^	50.88 ± 0.44 ^a^	42.98 ± 1.37 ^Bb^	46.27 ± 2.61 ^Ab^	44.31 ± 1.02 ^b^	45.78 ± 1.73 ^b^	43.67 ± 0.44 ^b^	44.55 ± 1.33 ^b^
PUFA	16.53 ± 1.01 ^c^	16.54 ± 1.31 ^b^	26.10 ± 0.85 ^Aa^	21.98 ± 2.61 ^Ba^	26.02 ± 1.31 ^a^	24.10 ± 2.03 ^a^	23.05 ± 0.54 ^b^	23.78 ± 1.68 ^a^
MUFA/SFA	1.53 ± 0.05 ^a^	1.56 ± 0.04 ^a^	1.39 ± 0.08 ^b^	1.46 ± 0.09 ^ab^	1.49 ± 0.04 ^a^	1.52 ± 0.08 ^ab^	1.31 ± 0.03 ^b^	1.41 ± 0.11 ^b^
PUFA/SFA	0.50 ± 0.03 ^c^	0.51 ± 0.05 ^b^	0.84 ± 0.03 ^Aa^	0.69 ± 0.08 ^Ba^	0.88 ± 0.06 ^a^	0.80 ± 0.08 ^a^	0.69 ± 0.03 ^b^	0.76 ± 0.09 ^a^

HH3, Hanhyup No.3; WRMD1, Woorimatdag No.1; WRMD2, Woorimatdag No.2. ^A,B^ Different letters represent a significant difference between fresh and frozen-thawed meat within the same breed (*p* < 0.05). ^a–c^ Different letters represent a significant difference between the fresh or frozen-thawed meat of different chicken breeds (*p* < 0.05). Mean ± SD.

**Table 6:** Comparison of volatile organic compounds of fresh and frozen-thawed chicken meats from Korean native chickens and broilers (A.U. ×10^6^).

VOCs	m/z	LRI	Broiler		HH3		WRMD1		WRMD2	
			Fresh	Frozen-Thawed	Fresh	Frozen-Thawed	Fresh	Frozen-Thawed	Fresh	Frozen-Thawed
**Acids**										
(E)-Hexadec-9-enoic acid	55	1942	0.000 ^b^	0.000	0.000 ^b^	0.000	0.016 ^a^	0.000	0.000 ^b^	0.000
Benzoic acid	122	1169	0.013	0.000	0.000	0.000	0.000	0.000	0.014 ^A^	0.000 ^B^
Dodecanoic acid	73.1	1564	0.000	0.000 ^b^	0.000	0.000 ^b^	0.000	0.000 ^b^	0.091	0.050 ^a^
Guanidineacetic acid	43	1069	0.000	0.117 ^a^	0.000	0.000 ^b^	0.000	0.000 ^b^	0.000	0.000 ^b^
n-Decanoic acid	73	1368	0.000	0.000 ^b^	0.000	0.000 ^b^	0.000	0.000 ^b^	0.000 ^B^	0.013 ^Aa^
n-Hexadecanoic acid	73	1963	0.446	0.095	0.600	0.075	0.373 ^A^	0.064 ^B^	0.355	0.171
Nonanoic acid	73.1	1274	0.000 ^b^	0.000 ^b^	0.033 ^Aab^	0.000 ^Bb^	0.000 ^b^	0.000 ^b^	0.078 ^a^	0.042 ^a^
Octadecanoic acid	73	2163	0.058	0.000	0.149	0.000	0.049 ^A^	0.000 ^B^	0.064	0.000
Octanoic acid	60.1	1178	0.000 ^B^	0.011 ^Ab^	0.000	0.000 ^b^	0.024 ^A^	0.000 ^Bb^	0.031	0.034 ^a^
Tetradecanoic acid	73	1761	0.039	0.008	0.027	0.012	0.036 ^A^	0.005 ^B^	0.040	0.027
**Subtotal**			0.556	0.230	0.808	0.087	0.497 ^A^	0.069 ^B^	0.673	0.337
**Alcohols**										
(S)-(+)-3-Methyl-1-pentanol	56.1	788	0.583 ^B^	1.802 ^Aa^	0.454	0.864 ^b^	0.461	0.320 ^b^	0.281	0.501 ^b^
1-Decanol, 2-ethyl-	57	1401	0.000	0.000 ^b^	0.000	0.000 ^b^	0.000 ^B^	0.129 ^Aa^	0.000 ^B^	0.060 ^Aab^
1-Dodecanol	57.1	1477	0.010	0.026	0.008	0.019	0.012	0.007	0.012	0.010
1-Heptanol	70.1	964	0.613 ^B^	2.718 ^Aa^	0.188	0.368 ^b^	0.242	0.080 ^b^	0.236	0.494 ^b^
1-Hexadecanol	83.1	1884	0.000 ^b^	0.000	0.000 ^b^	0.000	0.013 ^Aa^	0.000 ^B^	0.011 ^Aa^	0.000 ^B^
1-Hexanol, 2-ethyl-	57.1	1036	0.378	0.145	0.428 ^A^	0.133 ^B^	0.374 ^A^	0.131 ^B^	0.269 ^A^	0.075 ^B^
1-Hexanol, 5-methyl-2-(1-methylethyl)-	57	1065	0.120 ^Aab^	0.000 ^Bb^	0.173 ^a^	0.058 ^b^	0.000 ^Bb^	0.192 ^Aa^	0.000 ^b^	0.000 ^b^
1-Nonanol	56.1	1176	0.000 ^Bb^	0.062 ^Aa^	0.000 ^Bb^	0.024 ^Ab^	0.035 ^Aa^	0.015 ^Bb^	0.029 ^a^	0.016 ^b^
1-Octanol	56.1	1081	0.509 ^B^	1.834 ^Aa^	0.191	0.337 ^b^	0.302 ^A^	0.120 ^Bb^	0.341	0.456 ^b^
1-Octanol, 2-butyl-	71	1285	0.000	0.000 ^b^	0.000	0.014 ^ab^	0.000 ^B^	0.030 ^Aa^	0.000 ^B^	0.020 ^Aab^
1-Octen-3-ol	57	975	3.219 ^B^	14.784 ^Aa^	1.804	3.412 ^b^	2.310	1.479 ^b^	1.053	5.597 ^b^
2,4-Di-tert-butylphenol	191	1519	0.000	0.000 ^b^	0.000	0.000 ^b^	0.000	0.000 ^b^	0.000	0.020 ^a^
2-Hexyl-1-octanol	71.1	1601	0.000	0.000	0.000	0.010	0.000	0.012	0.000	0.000
2-Octen-1-ol, (E)-	57	1078	0.133	0.000 ^b^	0.119 ^A^	0.000 ^Bb^	0.105	0.070 ^a^	0.000	0.000 ^b^
2-Octen-1-ol, (Z)-	57.1	1078	0.000 ^B^	0.601 ^A^	0.063	0.156	0.103	0.000	0.000	0.306
5-Octen-2-ol, 5-methyl-	81	1049	0.000 ^B^	0.039 ^Aa^	0.000	0.000 ^b^	0.000	0.000 ^b^	0.000	0.000 ^b^
Cyclohexanol, 2,4-dimethyl-	81.1	1039	0.000 ^B^	0.091 ^Aa^	0.000	0.000 ^b^	0.000	0.000 ^b^	0.000	0.000 ^b^
Cyclohexanol, 5-methyl-2-(1-methylethyl)-	71	1176	0.184 ^Aa^	0.000 ^B^	0.181 ^Aa^	0.000 ^B^	0.000 ^b^	0.000	0.000 ^b^	0.000
Eugenol	164	1361	0.012 ^Aab^	0.000 ^B^	0.015 ^Aa^	0.000 ^B^	0.000 ^b^	0.000	0.000 ^b^	0.000
p-Cresol	107	1088	0.039 ^a^	0.042 ^a^	0.039 ^a^	0.037 ^a^	0.031 ^Aa^	0.013 ^Bb^	0.012 ^Ab^	0.000 ^Bb^
Phenol	94	984	0.035	0.000	0.026 ^A^	0.000 ^B^	0.000	0.000	0.000	0.000
Phenol, 2-methoxy-	124.1	1097	0.000 ^b^	0.000 ^b^	0.013 ^Aa^	0.006 ^Ba^	0.000 ^b^	0.000 ^b^	0.000 ^b^	0.000 ^b^
**Subtotal**			5.835 ^B^	22.143 ^Aa^	3.702	5.437 ^b^	3.988	2.596 ^b^	2.243	7.558 ^b^
**Aldehydes**										
2,4-Decadienal, (E,E)-	81	1320	0.035 ^Bab^	0.189 ^Aa^	0.000 ^b^	0.000 ^b^	0.059 ^Aa^	0.000 ^Bb^	0.000 ^b^	0.000 ^b^
2,4-Heptadienal, (E,E)-	81.1	1012	0.000 ^B^	0.102 ^Aa^	0.000	0.000 ^b^	0.000	0.000 ^b^	0.000	0.000 ^b^
2,4-Nonadienal, (E,E)-	81	1214	0.000 ^B^	0.145 ^Aa^	0.000	0.000 ^b^	0.000	0.000 ^b^	0.000	0.000 ^b^
2-Decenal, (E)-	70	1265	0.055 ^B^	0.170 ^Aa^	0.024 ^A^	0.000 ^Bb^	0.052 ^A^	0.000 ^Bb^	0.037	0.049 ^b^
2-Nonenal, (E)-	70.1	1164	0.042 ^B^	0.169 ^Aa^	0.029 ^A^	0.000 ^Bb^	0.035 ^A^	0.000 ^Bb^	0.030 ^A^	0.000 ^Bb^
2-Octenal, (E)-	55.1	1065	0.000 ^Bb^	0.478 ^Aa^	0.000 ^b^	0.000 ^b^	0.097 ^Aa^	0.000 ^Bb^	0.084 ^Aa^	0.000 ^Bb^
2-Undecenal	70	1366	0.044 ^B^	0.092 ^Aa^	0.028	0.029 ^b^	0.041 ^A^	0.005 ^Bb^	0.031	0.030 ^b^
5-Ethylcyclopent-1-enecarboxaldehyde	67	1033	0.033	0.155 ^a^	0.011 ^A^	0.000 ^Bb^	0.020	0.010 ^b^	0.014	0.000 ^b^
Benzaldehyde, 3,4-dimethyl-	134	1229	0.002 ^a^	0.000	0.000 ^b^	0.000	0.000 ^b^	0.000	0.000 ^b^	0.000
Benzeneacetaldehyde	91.1	1047	0.071 ^Ba^	0.145 ^Aa^	0.037 ^ab^	0.053 ^b^	0.070 ^a^	0.060 ^b^	0.026 ^b^	0.034 ^b^
Decanal	57.1	1206	0.111 ^Ba^	0.233 ^Aa^	0.078 ^ab^	0.070 ^b^	0.087 ^Aab^	0.042 ^Bb^	0.065 ^b^	0.060 ^b^
Dodecanal	57.1	1410	0.040	0.047 ^a^	0.034 ^A^	0.015 ^Bb^	0.036 ^A^	0.017 ^Bb^	0.030 ^A^	0.015 ^Bb^
Hexadecanal	82.1	1818	0.032 ^A^	0.000 ^Bb^	0.033	0.028 ^a^	0.038	0.031 ^a^	0.029 ^A^	0.000 ^Bb^
Hexanal, 5-methyl-	70.1	850	0.603 ^B^	3.305 ^Aa^	0.239	0.623 ^b^	0.351 ^A^	0.129 ^Bb^	0.294	0.668 ^b^
Nonanal	57.1	1113	1.511 ^B^	4.925 ^Aa^	0.656	1.373 ^b^	1.144 ^A^	0.641 ^Bb^	0.909	1.058 ^b^
Octanal	43.1	1003	0.513 ^B^	2.191 ^Aa^	0.204	0.464 ^b^	0.299 ^A^	0.126 ^Bb^	0.274	0.554 ^b^
Pentadecanal-	57	1717	0.043 ^A^	0.018 ^B^	0.059 ^A^	0.016 ^B^	0.074 ^A^	0.010 ^B^	0.057	0.026
Tetradecanal	57.1	1614	0.073 ^A^	0.023 ^B^	0.077 ^A^	0.016 ^B^	0.073 ^A^	0.000 ^B^	0.065 ^A^	0.022 ^B^
Tridecanal	57	1514	0.032 ^A^	0.019 ^Ba^	0.038 ^A^	0.011 ^Bab^	0.045 ^A^	0.000 ^Bb^	0.031 ^A^	0.013 ^Bab^
Undecanal	57	1312	0.017	0.020 ^a^	0.016 ^A^	0.000 ^Bb^	0.027 ^A^	0.000 ^Bb^	0.015 ^A^	0.000 ^Bb^
**Subtotal**			3.260 ^B^	12.424 ^Aa^	1.563	2.698 ^b^	2.547 ^A^	1.071 ^Bb^	1.991	2.529 ^b^
**Ester**										
2-Propenoic acid, 3-(4-methoxyphenyl)-, 2-ethylhexyl ester	178	2169	0.000 ^b^	0.000	0.000 ^b^	0.000	0.028 ^a^	0.000	0.000 ^b^	0.000
Arsenous acid, tris(trimethylsilyl) ester	207	712	18.288 ^A^	10.10 ^Ba^	15.105 ^A^	10.489 ^Ba^	17.836 ^A^	9.669 ^Bab^	14.363 ^A^	7.336 ^Bb^
Benzoic acid, 2-hydroxy-, ethyl ester	120	1274	0.018 ^A^	0.000 ^B^	0.054 ^A^	0.000 ^B^	0.017	0.000	0.017 ^A^	0.000 ^B^
Butylated Hydroxytoluene	205	1518	0.087 ^a^	0.047	0.063 ^Aab^	0.000 ^B^	0.075 ^Aab^	0.000 ^B^	0.030 ^Ab^	0.000 ^B^
Carbonic acid, decyl vinyl ester	57.1	1504	0.000	0.000	0.000 ^B^	0.022 ^A^	0.000	0.020	0.000	0.000
Carbonic acid, dodecyl vinyl ester	57	4	0.000	0.000 ^b^	0.000 ^B^	0.007 ^Aa^	0.000	0.000 ^b^	0.000	0.000 b
Decanoic acid, ethyl ester	88	1397	0.039 ^A^	0.000 ^B^	0.017 ^A^	0.000 ^B^	0.028	0.000	0.041 ^A^	0.000 ^B^
Diphosphoric acid, diisooctyl ester	83.1	941	0.000	0.331 ^a^	0.000	0.000 ^b^	0.000	0.000 ^b^	0.000	0.000 ^b^
Dodecanoic acid, ethyl ester	88.1	1596	0.024 ^Ab^	0.000 ^B^	0.000 ^b^	0.000	0.026 ^b^	0.000	0.100 ^Aa^	0.000 ^B^
Ethyl Oleate	55	1975	0.036 ^Aa^	0.000 ^B^	0.000 ^b^	0.000	0.000 ^b^	0.000	0.000 ^b^	0.000
Hexadecanoic acid, ethyl ester	88.1	1996	0.070 ^A^	0.000 ^B^	0.042 ^A^	0.000 ^B^	0.048 ^A^	0.000 ^B^	0.065 ^A^	0.000 ^B^
Hydrogen isocyanate	43	571	0.055	0.037	0.187	0.061	0.302	0.000	0.062	0.051
Methyl salicylate	120	1194	1.458	1.132 ^ab^	2.338	0.926 ^ab^	2.539	1.590 ^a^	1.235 ^A^	0.595 ^Bb^
n-Caproic acid vinyl ester	43	982	1.002 ^Ba^	3.929 ^Aa^	0.613 ^ab^	1.178 ^b^	1.118 ^a^	0.683 ^b^	0.275 ^b^	1.613 ^ab^
Octadecanoic acid, butyl ester	56	2389	0.039	0.000	0.079 ^A^	0.000 ^B^	0.057	0.000	0.058 ^A^	0.000 ^B^
Propanoic acid, 2-methyl-, 3-hydroxy-2,2,4-trimethylpentyl ester	71	1355	0.000 ^Bb^	0.102 ^A^	0.000 ^Bb^	0.141 ^A^	0.027 ^a^	0.081	0.016 ^Bab^	0.044 ^A^
Triisobutyl phosphate	99	1526	0.000	0.117	0.000	0.394	0.000	0.117	0.000	0.047
**Subtotal**			21.116 ^A^	15.796 ^Ba^	18.497 ^A^	13.218 ^Bab^	22.100 ^A^	12.159 ^Bab^	16.262 ^A^	9.686 ^Bb^
**Hydrocarbons**										
1-Hexene, 4-methyl-	57.1	650	0.000	0.000	0.000	0.000	0.000	0.000	0.000	0.019
1-Octadecyne	82	1820	0.000	0.000 ^b^	0.000	0.000 ^b^	0.000	0.000 ^b^	0.000 ^B^	0.009 ^Aa^
1-Pentene, 2-methyl-	56.1	592	0.000 ^b^	0.000	0.000 ^b^	0.000	0.000 ^b^	0.000	0.039 ^a^	0.000
2,4-Dimethyldodecane	57	1268	0.030 ^A^	0.000 ^B^	0.040	0.000	0.052 ^A^	0.000 ^B^	0.000	0.000
3,3-Dimethyl-1,2-epoxybutane	55.1	632	0.000	0.000 ^b^	0.000 ^B^	0.015 ^Aa^	0.000	0.000 ^b^	0.000	0.000 ^b^
Benzene, 1,2,3,5-tetramethyl-	119	1126	0.000	0.000 b	0.000	0.000 ^b^	0.000 ^B^	0.025 ^Aa^	0.000	0.000 ^b^
Benzene, 1,2,4,5-tetramethyl-	119	1121	0.035	0.032 ^a^	0.045 ^A^	0.013 ^Bab^	0.039	0.023 ^ab^	0.022 ^A^	0.008 ^Bb^
Benzene, 1,2,4-trimethyl-	105	985	0.000 ^Bc^	0.018 ^A^	0.030 ^Aa^	0.016 ^B^	0.000 ^Bc^	0.017 ^A^	0.010 ^Ab^	0.000 ^B^
Benzene, 1,3-bis(1,1-dimethylethyl)-	175.1	1258	0.254	0.326	0.370 ^A^	0.098 ^B^	0.332	0.284	0.213 ^A^	0.115 ^B^
Benzene, 1-ethyl-2,3-dimethyl-	119	10 92	0.000	0.000 ^b^	0.000	0.000 ^b^	0.000 ^B^	0.023 ^Aa^	0.000	0.000 ^b^
Benzene, 1-isocyano-3-methyl-	117	1144	0.026 ^Aa^	0.000 ^Bb^	0.014 ^ab^	0.012 ^a^	0.022 ^Aa^	0.010 ^Ba^	0.000 ^b^	0.000 ^b^
Benzene, 1-methyl-2-propyl-	105	1055	0.000	0.000	0.009	0.000	0.016	0.009	0.000	0.000
Benzene, 1-methyl-3-(1-methylethyl)-	119	1025	0.011	0.000	0.010 ^A^	0.000 ^B^	0.000	0.000	0.000	0.000
Benzothiazole	135	1223	0.000 ^b^	0.000	0.012 ^Aab^	0.000 ^B^	0.020 ^Aa^	0.000 ^B^	0.010 ^Ab^	0.000 ^B^
Butane, 2-azido-2,3,3-trimethyl-	57.1	606	20.162	0.000 ^b^	0.000	0.000 ^b^	0.000	2.834 ^a^	0.000	0.000 ^b^
Cyclooctane	83	937	0.000 ^B^	0.046 ^Aa^	0.000	0.000 ^b^	0.000	0.000 ^b^	0.000	0.000 ^b^
Cyclotetrasiloxane, octamethyl-	281	1009	30.123	19.632 ^ab^	35.997	30.1325 ^a^	28.512 ^A^	0.000 ^Bb^	35.764	19.431 ^ab^
Decane	57	999	0.574	0.329 ^ab^	0.454 ^A^	0.102 ^Bb^	0.615	0.520 ^a^	0.230	0.154 ^b^
Decane, 2,3,5,8-tetramethyl-	71	1299	0.000	0.000 ^b^	0.000	0.000 ^b^	0.000 ^B^	0.017 ^Aa^	0.000	0.000 ^b^
Decane, 2,4-dimethyl-	71	1116	0.026	0.021	0.038	0.006	0.037	0.019	0.019	0.008
Decane, 2,5,6-trimethyl-	57.1	1110	0.000	0.000 ^b^	0.000	0.000 ^b^	0.000	0.013 ^a^	0.000	0.000 ^b^
D-Limonene	93	1029	0.060 ^Aa^	0.013 ^Bb^	0.032 ^Ab^	0.012 ^Bb^	0.031 ^b^	0.118 ^a^	0.024 ^Ab^	0.011 ^Bb^
Dodecane	57	1200	0.537	0.423 ^a^	0.493 ^A^	0.152 ^Bbc^	0.576	0.380 ^ab^	0.323 ^A^	0.137 ^Bc^
Dodecane, 2,6,11-trimethyl-	71	1284	0.058	0.061 ^a^	0.071 ^A^	0.000 ^Bb^	0.079 ^A^	0.000 ^Bb^	0.046 ^A^	0.000 ^Bb^
Dodecane, 2-methyl-	57	1254	0.000	0.000 ^b^	0.000	0.000 ^b^	0.000 ^B^	0.012 ^Aa^	0.000	0.000 ^b^
Dodecane, 4,6-dimethyl-	71.1	1330	0.029	0.039 ^a^	0.029 ^A^	0.000 ^Bb^	0.031	0.000 ^b^	0.026 ^A^	0.000 ^Bb^
Dodecane, 4-methyl-	85	1268	0.000 ^b^	0.024	0.026 ^Aa^	0.003 ^B^	0.000 ^Bb^	0.004 ^A^	0.022 ^Aab^	0.000 ^B^
Dodecane, 5-methyl-	57.1	1248	0.000	0.000 ^b^	0.000	0.000 ^b^	0.000	0.000 ^b^	0.000	0.004 ^a^
Heptadecane	57	1702	0.048 ^A^	0.000 ^B^	0.053 ^A^	0.000 ^B^	0.055 ^A^	0.000 ^B^	0.055 ^A^	0.010 ^B^
Hexadecane	71.1	1600	0.213 ^A^	0.064 ^Ba^	0.229 ^A^	0.020 ^Bb^	0.249 ^A^	0.000 ^Bb^	0.249 ^A^	0.024 ^Bb^
Hexane, 3-ethyl-	43.1	772	0.110	0.079 ^b^	0.090	0.036 ^b^	0.098	0.219 ^a^	0.052	0.048 ^b^
Indane	117	1034	0.000	0.000 b	0.000 ^B^	0.001 ^Ab^	0.000 ^B^	0.005 ^Aa^	0.000	0.000 ^b^
Indole	117	1298	0.024 ^bc^	0.015 ^a^	0.054 ^Ab^	0.008 ^Bab^	0.122 ^Aa^	0.000 ^Bb^	0.003 ^c^	0.000 ^b^
Methane, dichloronitro-	83.1	590	0.161 ^B^	0.487 ^A^	0.086 ^B^	0.736 ^A^	0.105	0.154	0.064	0.622
Naphthalene	128	1181	0.046 ^A^	0.020 ^Ba^	0.056 ^A^	0.017 ^Bab^	0.049 ^A^	0.015 ^Bab^	0.045 ^A^	0.010 ^Bb^
n-Hexane	43.1	586	0.000 ^b^	0.000	0.218 ^Aab^	0.000 ^B^	0.000 ^b^	0.000	0.271 ^Aa^	0.000 ^B^
Nonane, 2,5-dimethyl-	57	1016	0.072	0.045 ^ab^	0.059	0.019 ^b^	0.067	0.072 ^a^	0.036	0.024 ^b^
Nonane, 2,6-dimethyl-	71	1026	0.101	0.077 ^ab^	0.100	0.032 ^b^	0.110	0.115 ^a^	0.051	0.039 ^b^
Nonane, 2-methyl-	57	952	0.043	0.033 ^a^	0.036 ^A^	0.000 ^Bb^	0.036 ^A^	0.000 ^Bb^	0.015	0.011 ^b^
Nonane, 4-methyl-	57	948	0.000	0.000 ^b^	0.000	0.000 b	0.000	0.011 ^a^	0.000	0.000 ^b^
Octane, 1,1’-oxybis-	71	1666	0.000 ^b^	0.000	0.000 ^b^	0.000	0.042 ^Aa^	0.000 ^B^	0.025 ^ab^	0.000
Octane, 2,4,6-trimethyl-	57.1	970	0.000	0.000 ^b^	0.000	0.000 ^b^	0.000 ^B^	0.047 ^Aa^	0.000	0.000 ^b^
Oxetane, 3-(1-methylethyl)-	42	654	1.068 ^B^	3.615 ^Aa^	0.461	0.867 ^b^	0.534	0.417 ^b^	0.305	1.039 ^b^
Oxetane, 3,3-dimethyl-	56.1	601	0.309	0.224 ^bc^	0.245	0.395 ^b^	0.408 ^B^	0.879 ^Aa^	0.357 ^A^	0.132 ^Bc^
Pentadecane	71	1499	0.115	0.074 ^a^	0.134 ^A^	0.000 ^Bb^	0.139 ^A^	0.000 ^Bb^	0.128 ^A^	0.000 ^Bb^
Pentadecane, 2-methyl-	57	1565	0.000 ^b^	0.000	0.023 ^Aa^	0.000 ^B^	0.000 ^b^	0.000	0.000 ^b^	0.000
Pentadecane, 3-methyl-	57	1572	0.024 ^A^	0.000 ^B^	0.030 ^A^	0.000 ^B^	0.022	0.000	0.030 ^A^	0.000 ^B^
Pentane, 3-methyl-	43.1	584	3.113 ^a^	0.000 ^b^	0.000 ^b^	0.000 ^b^	0.000 ^Bb^	0.349 ^Aab^	0.000 ^Bb^	0.373 ^Aa^
Tetradecane	57	1400	0.272	0.315 ^a^	0.343 ^A^	0.048 ^Bb^	0.332 ^A^	0.000 ^Bb^	0.281 ^A^	0.000 ^Bb^
Tetradecane, 2-methyl-	71	1466	0.000 ^b^	0.000	0.000 ^b^	0.000	0.000 ^b^	0.000	0.015 ^Aa^	0.000 ^B^
Tridecane	57.1	1304	0.245 ^A^	0.141 ^Ba^	0.264 ^A^	0.062 ^Bb^	0.262 ^A^	0.064 ^Bb^	0.209 ^A^	0.073 ^Bb^
Undecane	57.1	1109	0.080	0.060	0.066 ^A^	0.029 ^B^	0.080	0.031	0.050 ^A^	0.021 ^B^
Undecane, 2,3-dimethyl-	57.1	1264	0.000	0.000 ^b^	0.000	0.000 ^b^	0.000 ^B^	0.024 ^Aa^	0.000	0.000 ^b^
Undecane, 2,4-dimethyl-	85	1212	0.000	0.000 ^b^	0.000	0.000 ^b^	0.000 ^B^	0.006 ^Aa^	0.000	0.000 ^b^
Undecane, 2,5-dimethyl-	57	1215	0.000	0.000	0.039	0.000	0.000	0.000	0.000	0.000
Undecane, 2,6-dimethyl-	57.1	1215	0.030 ^Aa^	0.000 ^B^	0.000 ^B^	0.013 ^A^	0.000	0.016	0.017 ^ab^	0.013
Undecane, 2,8-dimethyl-	71.1	1224	0.015 ^A^	0.000 ^Bb^	0.022 ^A^	0.004 ^Bb^	0.020	0.011 ^a^	0.009 ^A^	0.000 ^Bb^
**Subtotal**			58.015	26.212	40.276	32.847	33.092 ^A^	6.743 ^B^	39.013	22.336
**Ketones**										
(+)-2-Bornanone	95	1148	0.000 ^b^	0.000	0.012 ^Aa^	0.000 ^B^	0.000 ^b^	0.000	0.000 ^b^	0.000
2-Butanone	43	586	0.000 ^Bb^	0.489 ^Aa^	0.000 ^Bb^	0.287 ^Aab^	0.873 ^a^	0.330 ^ab^	0.000 ^b^	0.000 ^b^
Acetophenone	105	1071	0.082 ^a^	0.043	0.074 ^a^	0.034	0.081 ^a^	0.043	0.000 ^Bb^	0.031 ^A^
Furan, 2-pentyl-	81.1	988	0.147 ^B^	0.375 ^Aa^	0.120 ^A^	0.000 ^Bb^	0.112 ^A^	0.000 ^Bb^	0.065	0.101 ^b^
1-Octen-3-one	55	973	0.000 ^B^	0.059 ^Aa^	0.000	0.000 ^b^	0.000	0.000 ^b^	0.000	0.000 ^b^
5-Hepten-2-one, 6-methyl-	43.1	985	0.000	0.000	0.042	0.000	0.052 ^A^	0.000 ^B^	0.063 ^A^	0.000 ^B^
5,9-Undecadien-2-one, 6,10-dimethyl-, (E)-	43	1456	0.043 ^A^	0.000 ^B^	0.024 ^A^	0.010 ^B^	0.080 ^A^	0.000 ^B^	0.025	0.008
N,N’-Bis(2,6-dimethyl-6-nitrosohept-2-en-4-one)	55	644	0.000 ^B^	0.018 ^Aa^	0.000	0.000 ^b^	0.000	0.000 ^b^	0.000	0.000 ^b^
**Subtotal**			0.273 ^Bb^	0.985 ^Aa^	0.273 ^b^	0.321 ^b^	1.198 ^Aa^	0.372 ^Bb^	0.153 ^b^	0.140 ^b^
**Others**										
1H-1,2,3,4-Tetrazol-5-ylmethanamine	43.1	1143	0.000	0.000	0.000	0.000	0.000	0.000	0.000	0.003
Arsine	76	579	0.000 ^b^	0.000	0.000 ^b^	0.000	5.367 ^Aa^	0.000 ^B^	0.000 ^b^	0.000
Camphor	95.1	1148	0.000 ^b^	0.000	0.014 ^Aa^	0.000 ^B^	0.000 ^b^	0.000	0.007 ^Aab^	0.000 ^B^
Cyclic octaatomic sulfur	64	2029	0.076 ^Ba^	0.132 ^Aa^	0.050 ^Bab^	0.109 ^Aab^	0.058 ^Bab^	0.111 ^Aab^	0.039 ^Bb^	0.063 ^Ab^
Formamide, N,N-dibutyl-	72	1308	0.000 ^Bc^	0.018 ^Aab^	0.033 ^b^	0.035 ^a^	0.106 ^Aa^	0.039 ^Ba^	0.046 ^Ab^	0.000 ^Bb^
Hexathiane	192	1493	0.018 ^a^	0.025 ^a^	0.010 ^Bab^	0.018 ^Aab^	0.000 ^Bb^	0.015 ^Ab^	0.008 ^ab^	0.016 ^b^
n-Butyl ether	57.1	860	0.000 ^b^	0.000 ^b^	0.117 ^Aa^	0.018 ^Ba^	0.000 ^b^	0.000 ^b^	0.000 ^b^	0.000 ^b^
sec-Butylamine	44.1	611	0.000 ^B^	2.184 ^Aa^	0.000	0.059 ^b^	0.000	0.198 ^b^	0.000	0.883 ^ab^
Subtotal			0.094 ^Bb^	2.359 ^Aa^	0.225 ^b^	0.239 ^b^	5.531 ^Aa^	0.364 ^Bb^	0.100 ^b^	0.965 ^ab^
**Total**			89.148	80.150 ^a^	65.344	54.848 ^ab^	68.953 ^A^	23.375 ^Bb^	60.435	43.551 ^ab^

HH3, Hanhyup No.3; WRMD1, Woorimatdag No.1; WRMD2, Woorimatdag No.2. ^A,B^ Different letters represent a significant difference between fresh and frozen-thawed meat within the same breed (*p* < 0.05). ^a^^–c^ Different letters represent a significant difference between the fresh or frozen-thawed meat of different chicken breeds (*p* < 0.05). LRI, Linear retention index.

**Table 7:** Comparison of sensory characteristics of fresh and frozen-thawed chicken meats from Korean native chickens and broilers.

Variables	Broiler		HH3		WRMD1		WRMD2	
	Fresh	Frozen-Thawed	Fresh	Frozen-Thawed	Fresh	Frozen-Thawed	Fresh	Frozen-Thawed
Color	7.67 ± 1.50	7.80 ± 0.77 ^a^	6.33 ± 1.45	7.00 ± 0.85 ^ab^	6.33 ± 1.76	6.53 ± 1.30 ^b^	7.20 ± 1.01	7.27 ± 0.56 ^ab^
Aroma	6.67 ± 1.50	7.13 ± 0.74	7.13 ± 1.19	7.47 ± 0.64	7.07 ± 1.71	7.27 ± 0.88	6.13 ± 1.46	7.00 ± 1.07
Taste	6.40 ± 1.45	7.13 ± 1.19	7.20 ± 1.26	7.20 ± 1.01	7.47 ± 1.41	7.27 ± 1.28	6.27 ± 0.96 ^B^	7.40 ± 1.24 ^A^
Flavor	6.40 ± 1.40 ^ab^	6.87 ± 1.13	6.93 ± 1.33 ^ab^	7.13 ± 0.92	7.47 ± 1.36 ^a^	7.20 ± 0.86	6.07 ± 1.10 ^B^^b^	7.27 ± 1.10 ^A^
Juiciness	6.53 ± 1.19 ^b^	7.20 ± 1.52	7.80 ± 0.94 ^Aa^	6.20 ± 1.66 ^B^	6.93 ± 1.44 ^ab^	6.73 ± 1.33	6.47 ± 1.55 ^b^	6.93 ± 1.16
Tenderness	6.27 ± 1.44 ^B^	7.73 ± 1.10 ^Aa^	7.13 ± 1.73	6.33 ± 1.18 ^b^	6.27 ± 1.71	7.00 ± 1.00 ^ab^	6.20 ± 1.61	7.13 ± 0.99 ^ab^
Texture	6.67 ± 1.40	6.80 ± 1.15	7.27 ± 1.62	7.00 ± 1.36	7.47 ± 1.25	7.27 ± 1.16	7.20 ± 1.52	7.47 ± 1.19
Overall acceptability	6.70 ± 1.25	6.77 ± 1.24	7.30 ± 1.13	6.77 ± 0.73	7.50 ± 1.32	7.10 ± 1.00	6.67 ± 1.05 ^B^	7.50 ± 0.98 ^A^

HH3, Hanhyup No.3; WRMD1, Woorimatdag No.1; WRMD2, Woorimatdag No.2. ^A,B^ Different letters represent a significant difference between fresh and frozen-thawed meat within the same breed (*p* < 0.05). ^a^^–c^ Different letters represent a significant difference between the fresh or frozen-thawed meat of different chicken breeds (*p* < 0.05).

## Data Availability

Data are available within the article.
